# Effects of Vitamin C on Organ Function in Cardiac Surgery Patients: A Systematic Review and Meta-Analysis

**DOI:** 10.3390/nu11092103

**Published:** 2019-09-04

**Authors:** Aileen Hill, Kai C. Clasen, Sebastian Wendt, Ádám G. Majoros, Christian Stoppe, Neill K. J. Adhikari, Daren K. Heyland, Carina Benstoem

**Affiliations:** 1 Department of Intensive Care Medicine, Medical Faculty RWTH Aachen, D-52074 Aachen, Germany; 2 Department of Anesthesiology, Medical Faculty RWTH Aachen, D-52074 Aachen, Germany; 3 3CARE—Cardiovascular Critical Care and Anesthesia Evaluation and Research, D-52074 Aachen, Germany; 4 Medical Student of the Medical Faculty RWTH Aachen University, D-52074 Aachen, Germany; 5 Department of Critical Care Medicine, Sunnybrook Health Sciences Centre, Interdepartmental Division of Critical Care Medicine, University of Toronto, Toronto, ON M4N 3M5, Canada; 6 Clinical Evaluation Research Unit, Kingston General Hospital, Kingston, CA K7L 2V7, Canada

**Keywords:** cardiac surgery, oxidative stress, reperfusion injury, antioxidant, vitamin, ascorbic acid, organ dysfunction, systematic review, meta-analysis

## Abstract

Background: Cardiac surgery is associated with oxidative stress and systemic inflammation, which both contribute to postoperative organ dysfunction. Vitamin C is a pleiotropic, antioxidant, and potentially organ-protective micronutrient. Past clinical trials and meta-analyses have focused predominantly on occurrence of postoperative atrial fibrillation. Therefore, we investigated the influence of perioperative vitamin C administration on clinically relevant parameters closer related to the patient’s recovery, especially organ function, and overall outcomes after cardiac surgery. Methods: Randomized controlled trials (RCTs) comparing perioperative vitamin C administration versus placebo or standard of care in adult patients undergoing cardiac surgery were identified through systematic searches in Pubmed, EMBASE, and CENTRAL on 23 November 2018. Published and unpublished data were included. Assessed outcomes include organ function after cardiac surgery, adverse events, in-hospital mortality, intensive care unit, and hospital length-of-stay. Data was pooled only when appropriate. Results: A total of 19 RCTs with 2008 patients were included in this meta-analysis. Vitamin C significantly decreased the incidence of atrial fibrillation (*p* = 0.008), ventilation time (*p* < 0.00001), ICU length-of-stay (*p* = 0.004), and hospital length-of-stay (*p* < 0.0001). However, on average, vitamin C had no significant effects on in-hospital mortality (*p* = 0.76), or on the incidence of stroke (*p* = 0.82). High statistical heterogeneity was observed in most analyses. Conclusions: Vitamin C impacts clinically and economically important outcomes, such as ICU and hospital length-of-stay, duration of mechanical ventilation and lowers the incidence of atrial fibrillation. Due to missing reports on organ dysfunction, this meta-analysis cannot answer the question, if vitamin C can improve single- or multiorgan function after cardiac surgery.

## 1. Introduction

Cardiac surgery triggers the release of inflammatory mediators and the production of reactive oxygen species, which lead to a systemic inflammatory response as well as oxidative stress [1,2]. The resulting disturbances in homeostasis contribute to the development of postoperative organ dysfunctions, which significantly determine the mid- to long-term outcome of the patients. In the past, several strategies have been developed to modulate and reduce the inflammatory responses, such as less invasive surgical techniques, leukocyte filters during cardiopulmonary bypass, and the use of immune-modulating drugs [3]. Despite these improvements, acute and persistent organ dysfunctions still occur frequently after cardiac surgery and consequently affect the patient’s outcome and quality of life [4,5].

Vitamin C is an essential and pleiotropic micronutrient that is involved in numerous processes in the human body. Vitamin C is a strong antioxidant, thereby countering the influence of free radicals and protecting the cells and organs from damage to macromolecules, such as cell membranes and DNA. Other crucial functions are vasopressor synthesis, restoration of vasopressor sensitivity, synthesis of collagen and carnitine, preservation of endothelial barriers and modulation of immune function [4,6,7,8,9,10].

A perioperative antioxidant treatment with vitamin C in patients undergoing cardiac surgery has been subject of investigation in several clinical trials and has consequently been analyzed in following meta-analyses. Surprisingly, most of them have focused predominantly on the occurrence of atrial fibrillation [11,12,13,14,15]. However, despite the clinical significance of postoperative arrhythmia, the effects of vitamin C on the patient’s organ functions and long-term outcomes remain largely unknown.

Therefore, we aimed to investigate the influence of vitamin C administration on clinically relevant parameters that are closer related to the patient´s recovery, especially organ function, and overall outcomes after cardiac surgery

## 2. Methods

This systematic review was registered at the PROSPERO international database under the registration number CRD42018115340. This systematic review was performed according to Cochrane Standard, the reporting will be in line with the PRISMA statement.

### 2.1. Search Strategy

We included randomized controlled trials (RCTs) comparing perioperative vitamin C administration versus placebo or standard of care in adult patients undergoing cardiac surgery. Relevant trials were identified through systematic searches of the databases PubMed, Embase, and Cochrane Central Register of Controlled Trials of the Cochrane Library (CENTRAL) on 23 November 2018. All publications published until November 2018 in at least one of these databases were included. The systematic search included the terms ”cardi*“, ”heart“, ”endocard*“, ”myocard*“, ”pericard*“, ”coronar*“, ”aort*“, ”bypass“, ”valv*“, ”vitamin c“, ”vit c“, ”ascorb*“ and ”surg*“, ”procedure“, ”operation“, ”intervention“ to be found in title or abstract. The following restrictions were made: ”clinical study”, ”clinical trial“, ”RCT“, ”review“, ”Cochrane Review“, ”Systematic Review“, ”Meta Analysis“, ”Controlled Clinical Trial“, ”Randomized Controlled Trial“ in the search masks of the according databases.

### 2.2. Study Selection Criteria

Studies were selected for inclusion in the review process if they met the following criteria:

- **Study design:** RCTs or meta-analysis of RCTs. Meta-analyses were reviewed for cross-referencing. When treatment allocation was not truly random, such as assigning a treatment intervention based on day of admission or month of service (pseudo-randomized trials), these trials were excluded.
- **Population:** Adult patients undergoing open heart surgery (with or without cardiopulmonary bypass)
- **Intervention:** Any form of perioperative vitamin C administration, defined by at least 1 day before, until 7 days after cardiac surgery. Studies with co-administration of other pharmacologic substances including pharmaconutrients and antioxidants were excluded, except for trials using the clinical standard treatment “ß-blockers” in both groups (see below).
- **Outcomes included in our meta-analysis:** Incidence of organ dysfunction, adverse events, intensive care unit (ICU), and hospital length-of-stay (LOS), hospital discharge location, and mortality

We included published and unpublished data. Reporting one or more of these outcomes in the trial was not an inclusion criterion for the review. Where a published trial did not report one of these outcomes, we accessed the trial protocol (if available) and contacted the trial authors to ascertain whether the outcomes were measured but not reported. We included relevant trials, which measured these outcomes but did not report the data at all, or not in a usable format, as part of the narrative.

After thorough discussion between all authors, it was decided to allow RCTs also administrating ß-blockers to the patients in both groups, as studies not using ß-blocker as co-intervention also reported high percentages of their patients receiving perioperative ß-blocker therapy. However, this complicates the interpretation of outcomes, especially regarding hemodynamics and perioperative arrhythmia. On the other hand, ß-blockers are clinical standard and including these patients in our meta-analysis will allow for easier translation into clinical practice.

### 2.3. Selection of Studies and Data Extraction

Of the identified potential studies, a database was constructed using the reference manager EndNote X9 (Clarivate Analytics, Boston, MA, USA). After identification and removal of duplicates, the titles and abstracts of the remaining studies were screened by two independent reviewers (AGM and KCC). Relevant full texts were retrieved and screened independently by two reviewers as well (AH and AGM) to select studies for inclusion, as well as to document reasons for exclusion of the ineligible studies. If there were any disagreements, a third author was asked to arbitrate (AH for abstract screening, KCC for full text screening). After abstract screening, authors of the selected studies were contacted for missing information. If essential information for the inclusion of the study was missing, the study was excluded. Full-texts published in a language other than English were translated or excluded if the full text could not be obtained (see Appendix A). Multiple reports of the same study were collated so that each study, rather than each report, is the unit of interest in the review.

### 2.4. Assessment of Risk of Bias in Included Studies

Two authors (AH, CB) independently assessed risk of bias for each study using the criteria outlined in the Cochrane Handbook for Systematic Reviews of Interventions [16]. Disagreements were solved by discussion. The results of the “risk of bias”-assessment were summarized in both a “risk of bias”-summary and a “risk of bias”-graph. Seven “risk of bias”-domains (random sequence generation (checking for possible selection bias); blinding of participants and personnel (checking for possible performance bias); blinding of outcome assessment (checking for possible detection bias); allocation concealment (checking for possible selection bias); incomplete outcome data (checking for possible attrition bias through withdrawals, dropouts, protocol deviations); selective reporting (checking for reporting bias); and other bias (checking for other biases)) were identified and assessed as recommended in the Cochrane Handbook of Interventions [16], see Appendix B for more detail.

### 2.5. Measures of Treatment Effect

Dichotomous data were analyzed as risk ratios (RR) with 95% confidence intervals (CI). For continuous data, mean difference (MD) with 95% CI for outcomes measured in the same way between trials were used as implemented in RevMan 5 (Reference Manager 5, Version 5.3, Nordic Cochrane Centre, The Cochrane Collaboration, 2014, Copenhagen, Denmark).

Investigators were contacted to verify key study characteristics and to obtain missing numerical outcome data where possible (e.g., when a study was identified as an abstract only). Analyses were carried out on an intention-to-treat basis for all outcomes, as far as possible. Statistical analysis was performed using RevMan 5. Meta-analyses were undertaken only where this was meaningful, that is, if the treatments, participants and the underlying clinical questions were similar enough for pooling to make sense. Given the clinical heterogeneity regarding our inclusion criteria (different types of open-heart surgery, different types of vitamin C administration) random-effects meta-analyses were used to produce an overall summary of average treatment effect across trials. The random-effects summary was treated as the average range of possible treatment effects and the clinical implications of treatment effects differing between trials were discussed. Results are presented as the average treatment effect with its 95% confidence interval, and the estimates of Tau^2^ and I^2^.

### 2.6. Assessment of Statistical Heterogeneity

Where data was pooled using meta-analysis, the presence of heterogeneity was assessed by visual inspection of forest plots and by examining the Chi^2^ test for [17]. Statistical heterogeneity was assessed in each meta-analysis using the Tau^2^, I^2^ and Chi^2^ statistics [18]. Heterogeneity was regarded substantial if:

1. The I^2^ value was high (exceeding 30%); and either:
2. There was inconsistency between trials in the direction or magnitude of effects (judged visually), or there was a low *p* value (<0.10) in the Chi^2^ test for heterogeneity; or
3. The estimate of between-study heterogeneity (Tau^2^) was above zero.

## 3. Results

### 3.1. Study Selection Process

**Figure 1:**
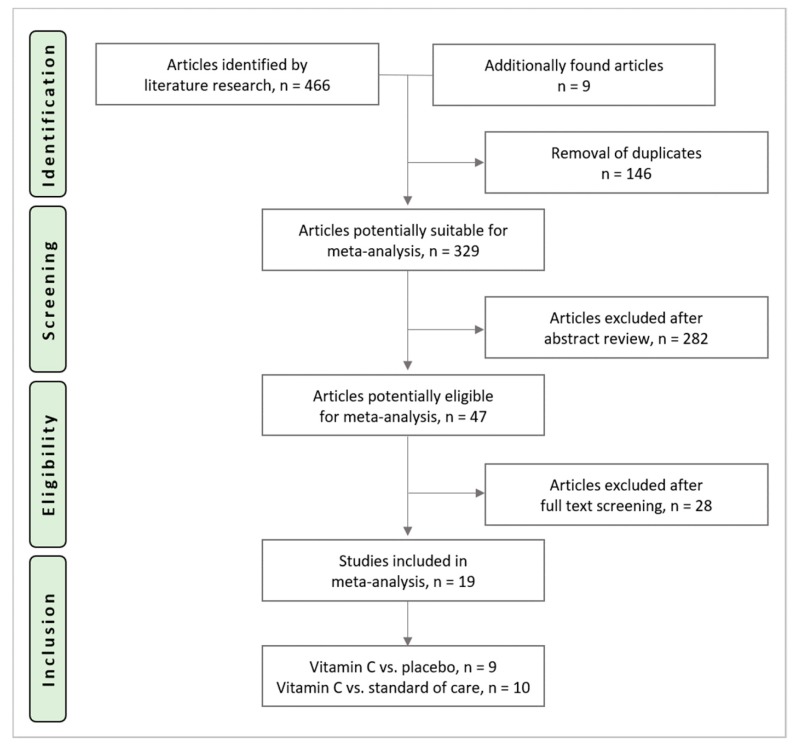
Study selection process.

The search identified 466 potential trials (PubMed: *n* = 149, Embase: *n* = 93, CENTRAL: *n* = 224). Nine additional articles were found during cross-referencing and from the authors own reference collections. After removal of 146 duplicates, 329 RCTs underwent title and abstract screening, 47 trials underwent full-text screening. Reasons for exclusion after full text screening are provided in Appendix A. Nineteen trials were included in this analysis and underwent data extraction. The study selection process is shown in Figure 1. 

### 3.2. Characteristics of Included Studies

Nineteen RCTs with 2008 patients were included. Their characteristics are described in Table 1. Nine trials including 1422 patients compared vitamin C to placebo and ten trials including 932 patients were controlled by standard of care. Eight studies including 724 patients administered the study medication intravenously and six studies including 734 patients administered orally. One study with 290 patients supplemented intravenously before and orally after surgery [19]. In four trials, the mode of administration was not available [20,21,22,23].

### 3.3. Risk of Bias Assessment

**Figure 2:**
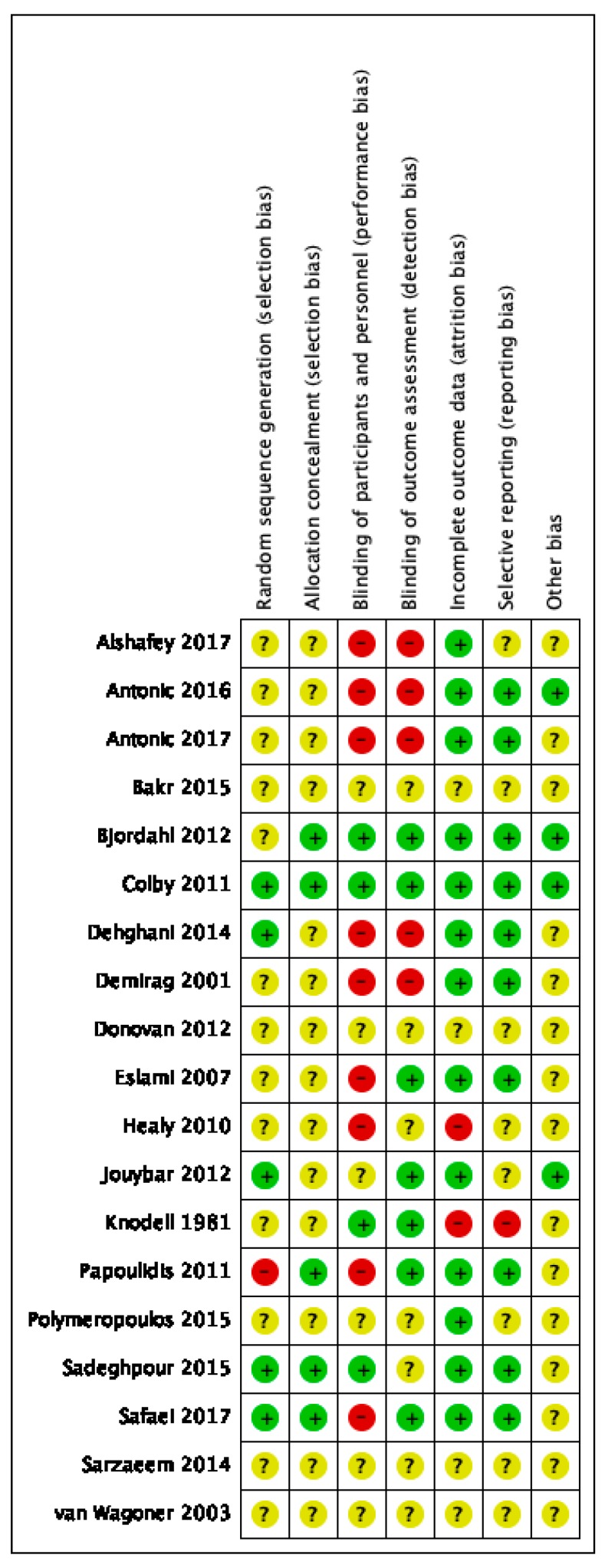
Risk of bias summary.

**Figure 3:**
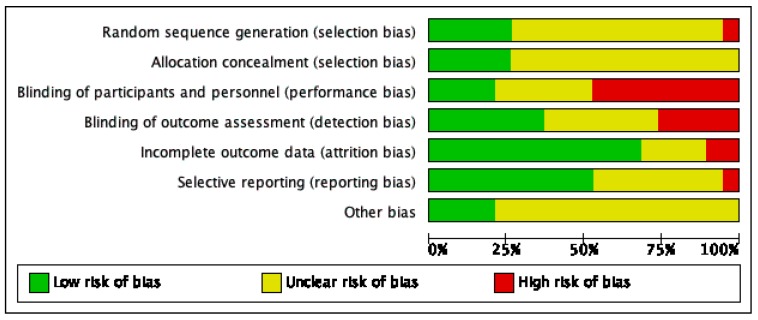
Risk of bias graph.

Risk of bias varied across included studies, and insufficient detail was provided to inform judgement in several included studies (see Figure 2 and Figure 3 for an overview and Supplementary Materials for full details).

## 4. Organ Function

Due to heterogeneity in outcome definition, measurement, and reporting, we were not able to perform meta-analysis on all endpoints; however, when pooling of data was not indicated, we provide a narrative overview to have an all-encompassing picture of the effects of vitamin C on organ function.

### 4.1. Neurologic Function

**Figure 4:**
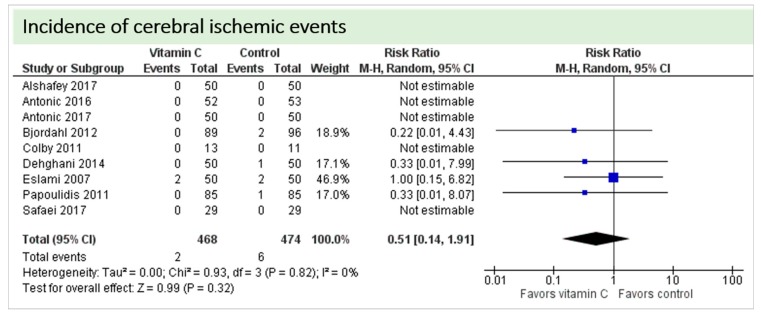
Total cerebral ischemic events.

No study reported on postoperative cognitive dysfunction or delirium. Nine studies including 942 patients reported on the occurrence of stroke as shown in Figure 4. In three of these, no stroke occurred in either group, and results were not estimable in meta-analysis. On average, no significant effect of vitamin C on the occurrence of stroke was observed (*p* = 0.82, CI 0.14 to 1.91) with a very low overall incidence of stroke (8/942 patients, 0.85%). No statistical heterogeneity was observed (I^2^ = 0%).

### 4.2. Cardiac Function

One study reported on postoperative vasopressor requirement and found a trend towards reduced number of patients requiring postoperative vasopressors in the vitamin C group (1/29 vs. 7/29 patients, *p* = 0.074) [36]. Another study reported on the duration of postoperative vasopressor treatment and observed no difference between the groups (1.1 ± 1.2 days [vitamin C] vs, 1.1 ± 1.3 days [control], *p* = 0.940) [26]. Two studies reported on use of inotropes during the first 24 h after surgery, which was significantly lower in the control group (56% vitamin C group vs. 36% in the control group, *p* = 0.045) in Alshafey´s RCT [20] and similar (20% in the vitamin C group vs. 22% in the control group, *p* = 0.81) in the RCT by Eslami et al. [31]. No study reported on cumulative or maximum vasopressor dosage as surrogate parameters of cardiac dysfunction.

**Figure 5:**
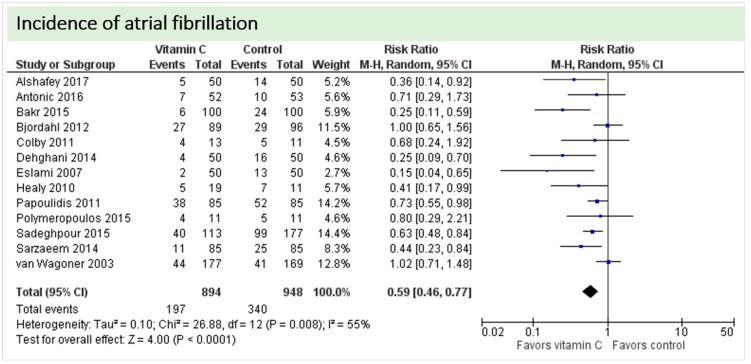
Incidence of postoperative atrial fibrillation.

One study reported on postoperative ejection fraction, which was significantly higher in patients receiving vitamin C either intravenously or orally administered as grape seed extract (49.7 ± 1.0 vs. 41.7 ± 1.5, *p* = 0.002) [36]. One study described similar hemodynamic parameters including systolic and diastolic blood pressure and heart rate in both groups (*p* = 0.37) [32]. Thirteen studies including 1842 patients reported on atrial fibrillation in the postoperative period. On average, a significant effect in favor of vitamin C was observed (*p* = 0.006, CI 0.46 to 0.77), Figure 5. High statistical heterogeneity was observed (I^2^ = 55%). In addition, one study reported arrhythmias requiring treatment, which was not significantly different between the groups (2/29 in the vitamin C group vs. 6/29 in the control group, *p* = 0.31) [36].

### 4.3. Pulmonary Function

**Figure 6:**
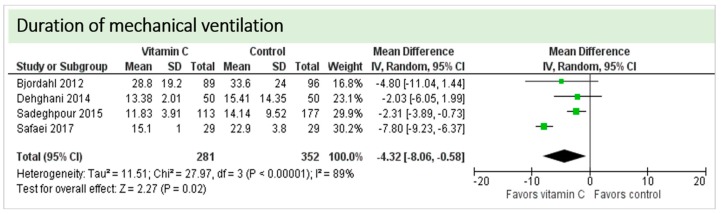
Duration of mechanical ventilation in hours.

No study reported on need for non-invasive ventilator support, oxygen requirement or pleural effusions as surrogate parameters of pulmonary function. Four studies including 633 patients reported on ventilation time in hours as shown in Figure 6. Two studies reported results in favor of vitamin C, two studies were neutral. On average, the effect of vitamin C was significant on reduction of ventilation time (*p* < 0.00001, CI −8.06 to −0.58); we observed high statistical heterogeneity (I^2^ = 89%). The effect of vitamin C was more pronounced in studies with intravenous administration of vitamin C [19,36]. One study reported a significantly higher percentage of patients weaned from mechanical ventilation within the first 24 h (72% in the vitamin C group vs. 66% in the control group, *p* = 0.048) [20].

### 4.4. Renal Function

Two studies including 127 patients reported on acute kidney injury [25,36]. In the study by Antonic et al., vitamin C had no effect on occurrence of acute kidney injury (*p* = 0.779), maximum postoperative serum creatinine (*p* = 0.434) or lowest postoperative creatinine clearance (*p* = 0.766). No patient received renal replacement therapy in either group in this study [25]. The study by Safaei et al. reported no acute kidney failure in either study groups (each 0/29) [36]. One study described “similar serum potassium, blood urea nitrogen and creatinine levels” in both groups, without presenting more detailed information [32]. Overall only insufficient data were available about the exact definition of acute kidney injury in these patients.

### 4.5. Adverse Events

Adverse events (as defined by trial authors) included stroke, sternal wound infection, sepsis, cardiac arrest, “some complications” (death, impaired renal function and infection), need for intra-aortic balloon pump, posttransfusion hepatitis, reoperation, and drainage volume. Pooling of data in meta-analysis was deemed as inappropriate due to the heterogeneity in outcome definition, measurement and reporting.

### 4.6. In-Hospital Mortality

**Figure 7:**
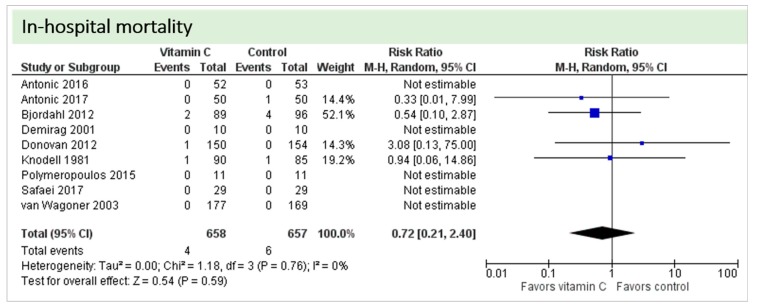
In-hospital mortality.

Nine studies including 1315 patients reported on this outcome. In five studies, no deaths occurred, and data could not be estimated. On average, no significant effect of vitamin C was found on in-hospital mortality (*p* = 0.72, CI 0.21 to 2.40), as shown in Figure 7. One study described two in-hospital deaths but did not specify the groups of the respective patients [24]. No statistical heterogeneity was observed (I^2^ = 0%).

### 4.7. Length-Of-Stay

**Figure 8:**
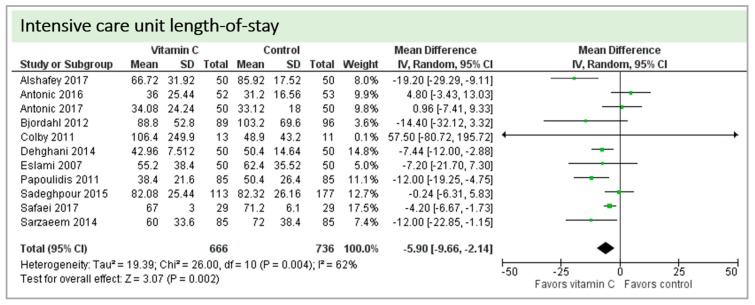
ICU length-of-stay.

Eleven Studies including 1402 patients reported on ICU-LOS as shown in Figure 8. One study reported longer overall ICU-LOS than all other RCTs [27]. On average, a significant effect favoring vitamin C administration was detected (*p* = 0.004, CI −9.66 to −2.14). High statistical heterogeneity was observed (I^2^ = 62%).

**Figure 9:**
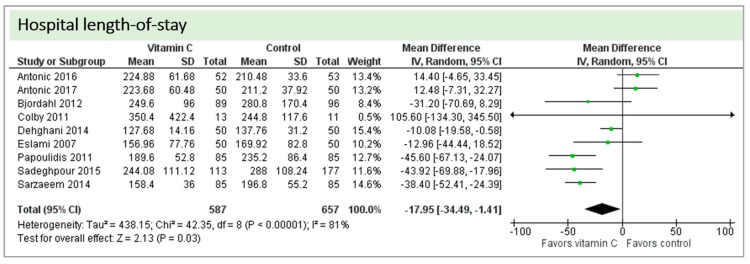
Hospital length-of-stay.

Eight studies including 1244 patients reported on hospital-LOS. The study by Colby et al. [27] reported much longer hospital stays than the other studies. On average, there was a significant effect in favor of vitamin C (*p* < 0.00001, CI −34.49 to −1.41). We observed high statistical heterogeneity (I^2^ = 81%) (Figure 9).

### 4.8. Subgroup Analysis Influence of Administration Route: Intravenous Administration versus Oral Administration of Vitamin C

This subgroup analysis was performed to investigate any possible influence of the route of administration (intravenous administration versus oral administration of vitamin C) on study results; available data allowed assessment of the outcomes cerebral ischemic events, incidence of atrial fibrillation, in-hospital mortality, ICU-LOS, hospital-LOS, and duration of mechanical ventilation.

**Figure 10:**
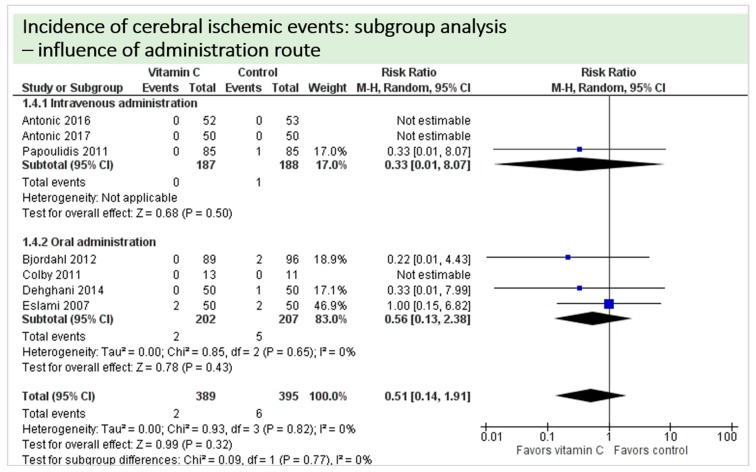
Cerebral ischemic events: subgroup analysis—influence of administration route.

A total of eight studies contributed to the subgroup analysis investigating any possible influence of the route of administration on the outcome “cerebral ischemic events”, as shown in Figure 10. The evidence suggested no difference in treatment effect for this subgroup, as treatment effects did not reach statistical significance in either group.

**Figure 11:**
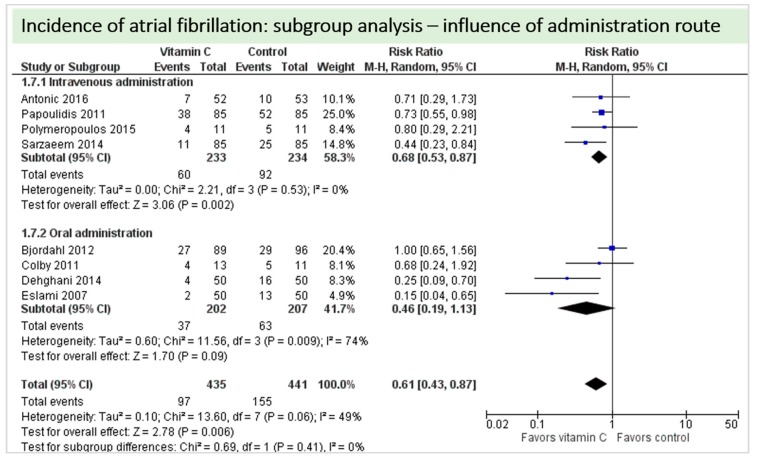
Incidence of atrial fibrillation: subgroup analysis—influence of administration route.

A total of eight studies contributed to the subgroup analysis investigating any possible influence of the route of administration on the outcome incidence of “atrial fibrillation”, as shown in Figure 11. While the effect of the treatment was statistically significant in the group receiving intravenous vitamin C (*p* = 0.002, CI 0.53 to 0.87, I^2^ = 0%), it was not in patients receiving oral vitamin C (*p* = 0.06, CI 0.19 to 1.13, I^2^ = 74%).

**Figure 12:**
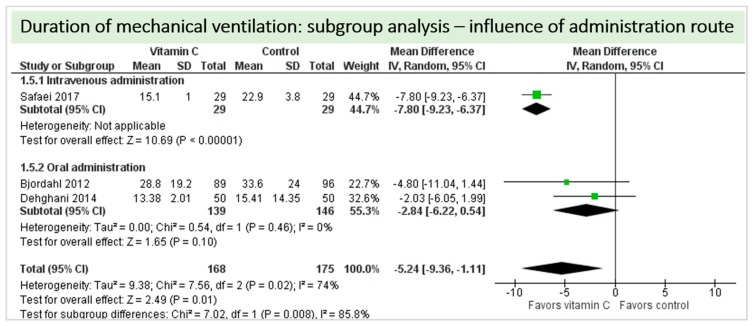
Duration of mechanical ventilation in hours: subgroup analysis—influence of administration route.

A total of three studies contributed to the subgroup analysis investigating any possible influence of the route of administration on the outcome “duration of mechanical ventilation”, as shown in Figure 12. We found a statistical significance in the group receiving intravenous vitamin C (*p* < 0.00001, CI−9.23 to −6.37, I^2^ not applicable); however, this group included only one RCT with 58 patients in total. In the group of oral vitamin C administration, the treatment effect did not reach statistical significance (*p* = 0.10, CI −6.22 to 0.54, I^2^ = 0%).

**Figure 13:**
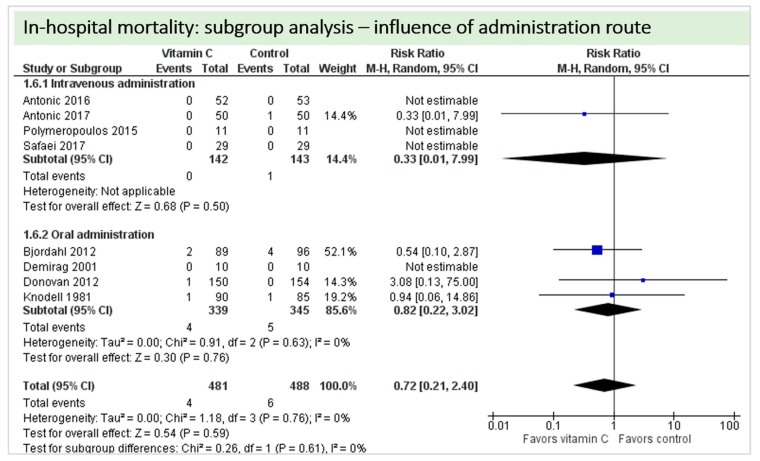
In-hospital mortality: subgroup analysis—influence of administration route.

A total of eight studies contributed to the subgroup analysis investigating any possible influence of the route of administration on the outcome “in-hospital mortality”, as shown in Figure 13. We found no evidence of a treatment effect between subgroups, as the treatment effect was not statistically significant in either group.

**Figure 14:**
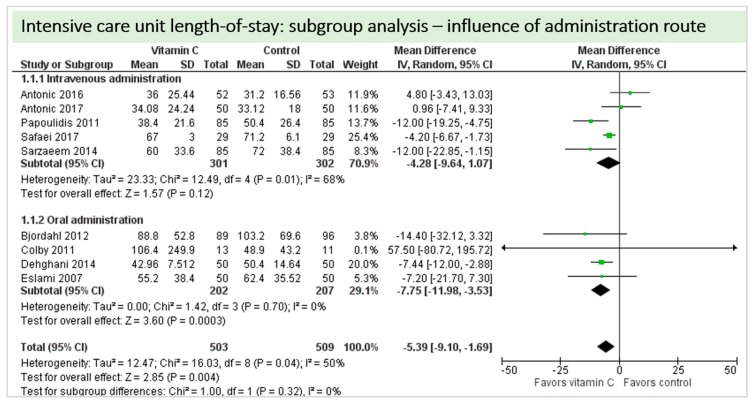
ICU length-of-stay: subgroup analysis—influence of administration route.

A total of nine studies contributed to the subgroup analysis investigating any possible influence of the route of administration on the outcome “ICU-LOS”, as shown in Figure 14. We found no statistically significant effects in the group receiving intravenous vitamin C (*p* = 0.12, CI −9.64 to 1.07, I^2^ = 68%), but in the group of oral vitamin C administration, the treatment effect did reach statistical significance (*p* = 0.0003, CI −11.98 to −3.53, I^2^ = 0%).

**Figure 15:**
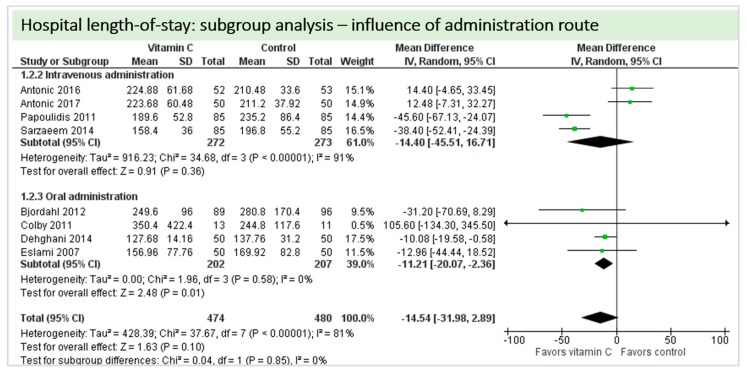
Hospital length-of-stay: subgroup analysis—influence of administration route.

A total of eight studies contributed to the subgroup analysis investigating any possible influence of the route of administration on the outcome “hospital-LOS”, as shown in Figure 15. We found no statistical significance in the group receiving intravenous vitamin C (*p* = 0.36, CI−45.51 to 16.71, I^2^ = 91%), but in the group of oral vitamin C administration, the treatment effect did reach statistical significance (*p* = 0.01, CI −20.07 to −2.36, I^2^ = 81%).

### 4.9. Subgroup Analysis Influence of Control Group: “Vitamin C versus Placebo” versus “Vitamin C versus Standard of Care”

**Figure 16:**
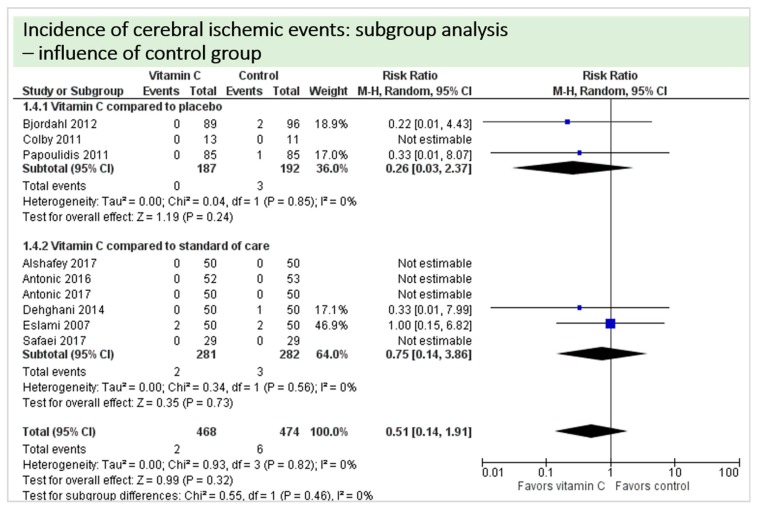
Cerebral ischemic events: subgroup analysis—influence of control group.

A total of eight studies contributed to the subgroup analysis investigating any possible influence of the control group on the outcome “cerebral ischemic events”, as shown in Figure 16. We found no evidence of a treatment effect between subgroups, as treatment effect did not reach statistical significance in either group.

**Figure 17:**
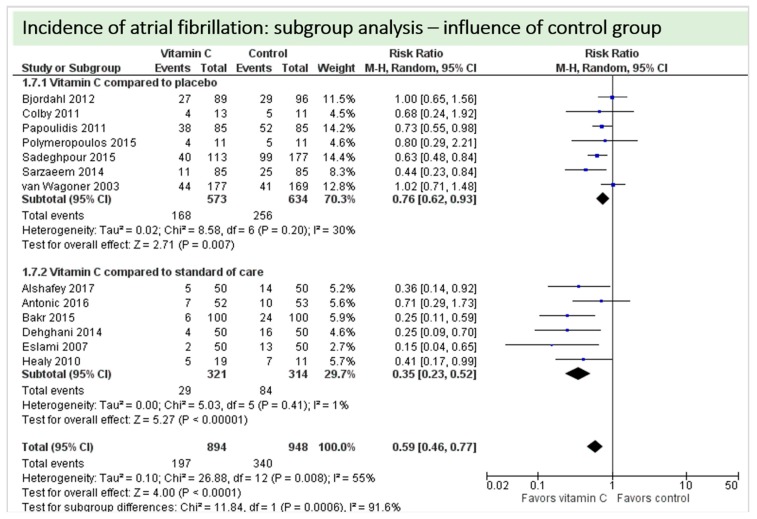
Incidence of atrial fibrillation: subgroup analysis—influence of control group.

A total of thirteen studies contributed to the subgroup analysis investigating any possible influence of the control group on the outcome incidence of “atrial fibrillation”, as shown in Figure 17. We found no evidence of a treatment effect between subgroups, as the treatment effect was significant in both groups.

**Figure 18:**
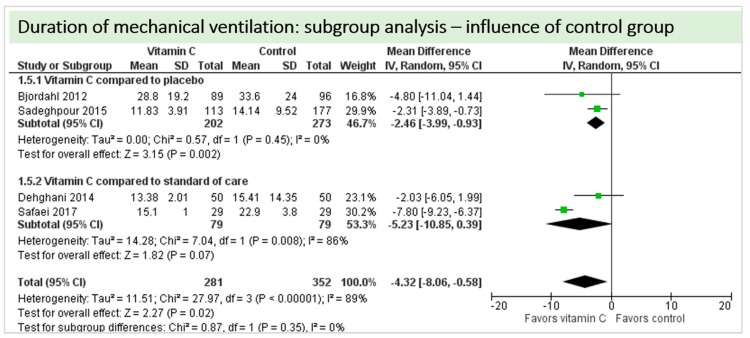
Duration of mechanical ventilation in hours: subgroup analysis—influence of control group.

A total of four studies contributed to the subgroup analysis investigating any possible influence of the control group on the outcome “duration of mechanical ventilation”, as shown in Figure 18. We found a statistical significance in the group comparing vitamin C to placebo (*p* = 0.002, CI −3.99 to –0.93, I^2^ = 0%), but not in the group comparing vitamin C to standard of care (*p* = 0.07, CI −10.85 to 0.39, I^2^ = 86%).

**Figure 19:**
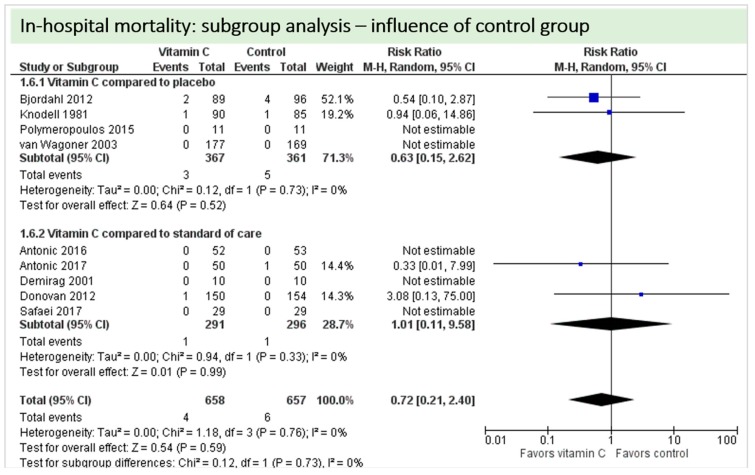
Mortality: subgroup analysis—influence of control group.

A total of nine studies contributed to the subgroup analysis investigating any possible influence of the control group on the outcome “in-hospital mortality”, as shown in Figure 19. We found no evidence of a treatment effect between subgroups, as the treatment effect was not statistically significant in either group.

**Figure 20:**
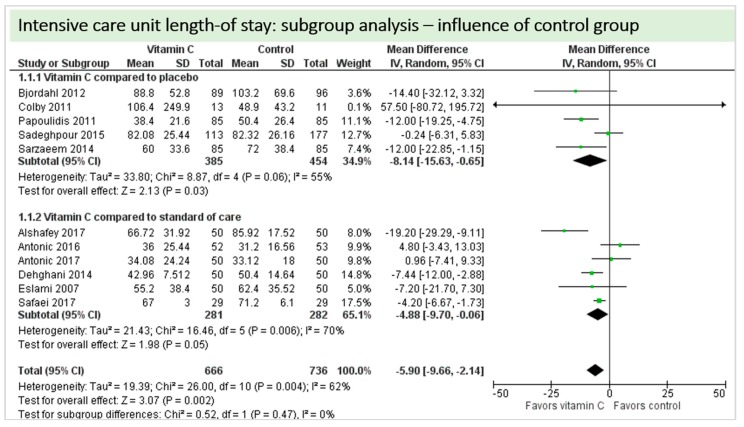
ICU length-of-stay: subgroup analysis—influence of control group.

A total of eleven studies contributed to the subgroup analysis investigating any possible influence of the control group on the outcome “ICU-LOS”, as shown in Figure 20. We found no evidence of a treatment effect between subgroups, as the treatment effect was significant in both groups.

**Figure 21:**
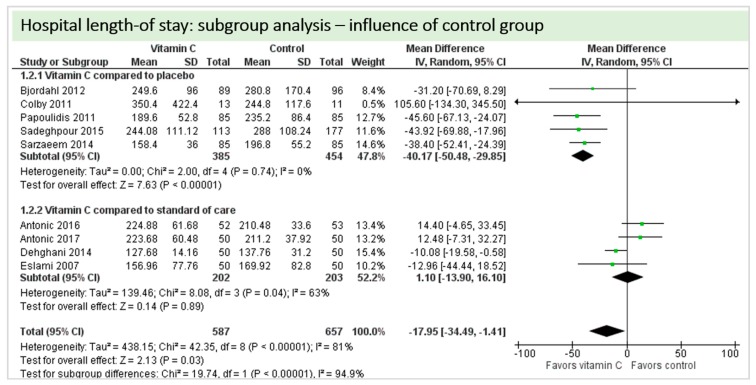
Hospital length-of-stay: subgroup analysis—influence of control group.

A total of eight studies contributed to the subgroup analysis investigating any possible influence of the control group on the outcome “hospital-LOS”, as shown in Figure 21. We found a statistical significance in the group receiving intravenous vitamin C (*p* < 0.00001, CI −50.48 to −29.85, I^2^ = 0%). In the group of oral vitamin C administration, the treatment effect did not reach statistical significance (*p* = 0.89, CI −13.90 to 16.10, I^2^ = 63%)

### 4.10. Sensitivity Analysis

Sensitivity analysis by limiting analyses to studies at low risk of bias for sequence generation, allocation concealment and incomplete outcome data for the primary outcome were not performed, as matching studies did not fulfil the corresponding criteria to allow pooling to make sense.

## 5. Discussion

Nineteen RCTs with 2008 patients, comparing the perioperative administration of vitamin C with placebo or standard of care in adult patients undergoing cardiac surgery, were systematically identified and data were aggregated for meta-analysis where appropriate.

On average, no significant effect of vitamin C was found on in-hospital mortality. Regarding our main outcome—organ dysfunction—vitamin C had no influence on the occurrence of stroke. However, vitamin C significantly decreased the incidence of atrial fibrillation and ventilation time as a marker of pulmonary dysfunction. Various adverse events were reported, but the heterogeneity limited the pooling of data. Regarding renal dysfunction, there was not enough data to pool.

Overall, only few studies reported on postoperative organ dysfunction. Reports were mostly limited to a single disease or surrogate parameter of one organ system, sometimes with very low overall incidences.

### 5.1. Quality of the Evidence

Risk of bias varied across included studies, and insufficient detail was provided to inform judgement in several cases. Three studies were reported as abstracts only, for two studies we had to rely on data from clinical trial registries only, although studies were completed several years ago. We have contacted several authors, sometimes several from one publication, but had a very low response rate to verify key characteristics and missing results of included studies. Especially with regard to random sequence generation and allocation concealment, the majority of studies either did not perform, or did not report both key aspects for selection bias adequately. We regard both measures against selection bias as key principles of RCTs and we want to highlight the importance of improving clinical trial methodology in future trials regarding this aspect—an aim that can be achieved easily with freely accessible and available methods. About blinding of personnel, patients, or outcome assessor, here too, studies have found it difficult to implement this. However, possible effects of these difficulties on performance or detection bias considering the assessed outcome parameters have to be discussed cautiously. We would like to highlight that the applicability of our results should be interpreted based on the described limitations of the quality of the available evidence.

Given the clinical heterogeneity regarding our inclusion criteria (different types of open-heart surgery, different types of vitamin C administration), random-effects meta-analyses were used to produce an overall summary of average treatment effect across trials. The correctness of this decision was reflected by the existence of statistical heterogeneity in the majority of the meta-analyses. Here, heterogeneity in outcome definition, measurement, and reporting limited the possibility to perform meta-analyses and, as a result, the overall quality of the available evidence.

We performed two independent subgroup analyses (subgroup analysis influence of administration route: Intravenous administration versus oral administration of vitamin C; and subgroup analysis influence of control group: “vitamin C versus placebo” versus “vitamin C versus standard of care”) to explain statistical heterogeneity among study results. With regard to the subgroup analysis influence of administration route, we found evidence of a treatment effect between subgroups for the outcomes “atrial fibrillation, ICU- and hospital-LOS”. With regard to the subgroup analysis influence of control group “vitamin C versus placebo” versus “vitamin C versus standard of care”, we found evidence of a treatment effect between subgroups for the outcomes “mechanical ventilation and hospital-LOS”. Differences in dosages and route of action of vitamin C when applied intravenously or orally explains the presence of statistical heterogeneity across trials. However, with regard to a possible influence of the control group, the presence of statistical heterogeneity cannot be assessed conclusively.

### 5.2. Potential Biases in the Review Process

We undertook this systematic review in accordance with the Cochrane Handbook for Systematic Reviews of Interventions and followed the guidance of the PRISMA statement for reporting of systematic reviews. We carried out a wide-ranging search across relevant electronic databases and clinical trial registries. We assessed reference lists of included studies and we have described the process of study selection methodically and in full detail. In addition, we screened reference lists of systematic reviews and contacted study authors repeatedly for additional data when needed. We did not apply any language or date restrictions and obtained translations by native speakers. Two review authors performed all steps of the selection process independently and analyses were conducted by one reviewer and checked by a colleague. Furthermore, we gave reasons why a study was not included in our analysis. We described each included study and made explicit judgements about whether studies were at low or high risk of bias. As a result, we identified no other potential sources of bias with regard of the conduct of this systematic review.

### 5.3. Agreements and Disagreements with Other Reviews

The results of our meta-analyses are confirmed by other published meta-analyses investigating the effect of vitamin C in cardiac surgery patients, which mainly focus on the rate of atrial fibrillation. A reduced ICU-LOS was also observed by three meta-analyses focusing exclusively on perioperative vitamin C administration [11,13,15] and one meta-analysis investigating the antioxidant effects of vitamins by Geng et al. [38]. Four meta-analyses also found significantly shorter hospital-LOS associated with vitamin C [11,15,39,40]. However, Hu et al. performed a meta-analysis where vitamin C administration was not associated with reductions in either ICU- or hospital-LOS [14].

Regarding atrial fibrillation, our results confirm the results of other meta-analyses [12,13,14,15], who all found a significant beneficial effect of vitamin C. None of the other meta-analyses assessed organ dysfunctions other than atrial fibrillation, but Shi et al. found significantly fewer adverse events in patients receiving vitamin C [15].

### 5.4. Implications for Practice

Postoperative organ dysfunctions in patients after cardiac surgery are associated with a complicated postoperative course. Despite substantial improvements in the surgical techniques, perfusion, and anesthesiologic management, the incidence and morbidity of patients undergoing cardiac surgery remains high, especially in those patients with combined or complex procedures [41]. This necessitates more effective strategies to counteract the frequently observed inflammatory response, which leads to the development of organ dysfunctions. Among these, the perioperative administration of vitamin C has emerged a promising strategy which has been evaluated in several clinical studies and analyzed in the present meta-analysis. The high heterogeneity between the included RCTs limit this meta-analysis and the applicability for the clinical routine.

While perioperative vitamin C administration seems to be beneficial regarding several outcomes, this meta-analysis cannot answer the question, if vitamin C can prevent single- or multiorgan dysfunction after cardiac surgery.

### 5.5. Implications for Research

Future trials should focus in greater detail on outcomes influencing patient-relevant and long-term outcomes, such as quality of life, organ dysfunction, discharge location, or return-to-work capability. These clinically relevant outcomes, including surrogate parameters of the dysfunction of the different organ systems, should be reported in greater detail, as they might be more sensitive to assess the effectivity of antioxidants or other micronutrients.

The observed heterogeneity between trials regarding population, timing, dosage and route of administration, as well as reported outcomes leading to difficulties in pooling the data for meta-analysis. This implies the need for the application of core measurement and core outcome sets for micronutrient administration in patients undergoing major surgery.

Both the rather low mortality rates and low incidence of postoperative organ dysfunctions indicated that the majority of included studies evaluated the effects of vitamin C on a rather low-risk group of cardiac surgery patients. In contrast, only few studies were available which focused only on patients with complex procedures and resulting prolonged ICU-LOS, which are most vulnerable for postoperative complications and thus of high interest for specific anti-inflammatory strategies, such as vitamin C.

## 6. Conclusions

Vitamin C impacts clinically and economically important outcomes, such as ICU- and hospital length-of-stay, and duration of mechanical ventilation, and lowers the incidence of atrial fibrillation. Due to missing reports on organ dysfunction, this meta-analysis cannot answer the question of if vitamin C can improve single- or multiorgan function after cardiac surgery. Future RCTs should focus on the selection of a patient group more vulnerable to a prolonged ICU stay, as well as on careful reports on clinically- and patient-relevant outcomes.

## Figures and Tables

**Table 1:** Characteristics of included studies, CABG = coronary artery bypass graft, CPB = cardiopulmonary bypass, Vit C = Vitamin C, i.v. = intravenously, p.o. = orally, preop = before surgery, postop = after surgery, n.a. = not available.

	Author and Year	Patients	Dosage and Timing of Vitamin C in the Intervention Group	Route	Control Group
**1**	**Alshafey 2017** [20]	100 scheduled CABG	Preop: 2 g daily for at least 3 days pre-operatively	n.a.	Standard of care
**2**	**Antonic 2016** [24]	105 elective CABG with CPB	Preop: 2 × 2 g: 24 and 2 h before surgery Postop: 2 × 1 g/day for 5 days	i.v.	Standard of care
**3**	**Antonic 2017** [25]	100 elective CABG with CPB	Preop: 2 × 2 g: 24 and 2 h Postop: 2 × 1 g/day for 5 days	i.v.	Standard of care
**4**	**Bakr 2015** [21]	200 CABG	Preop: at least one week, dosage and route not specified	n.a.	Standard of care
**5**	**Bjordahl 2012** [26]	185 scheduled CABG	Preop: 1 × 2 g night before surgery Postop: 2 × 1 g/day for 5 days	p.o.	Placebo
**6**	**Colby 2011** [27]	24 scheduled CABG and/or valvular surgery	Preop: 1 × 2 g night before Postop: 2 × 0.5 g/day for 4 days	p.o.	Placebo
**7**	**Dehghani 2014** [28]	100 elective isolated CABG with CPB	Preop: 1 × 2 g before the surgery Postop: 2 × 0.5 g/day for 5 days	p.o.	Standard of care
**8**	**Demirag 2001** [29]	30 elective CABG	Group 1: 2 × 50 mg/kg vitamin C after induction and before declamping	i.v.	Standard of care
**9**	**Donovan 2012** [30]	150	Preop: 2 g the morning before surgery Postop: 2 × 1 g for 5 days	p.o.	Standard of care
**10**	**Eslami 2007** [31]	100 elective isolated CABG patients with CPB	Preop: 2 g the night before surgery Postop: 2 × 1 g for 5 days	p.o.	Standard of care
**11**	**Healy 2010** [22]	60 CABG and/or valve in interim analysis	n.a.	n.a.	Standard of care
**12**	**Jouybar 2012** [32]	40 elective CABG	Preop: 2 × 3 g 12–18 h before surgery and after induction of anesthesia	i.v.	Placebo
**13**	**Knodell 1981** [33]	175 elective cardiac surgery	Preop: 4 × 800 mg/day for 2 days Postop: 4 × 800 mg/day for 2 weeks, started as soon as patient could take oral liquids	p.o.	Placebo
**14**	**Papoulidis 2011** [34]	170 elective isolated CABG with CPB	Preop: 1 × 2 g 3 h prior to initiation of CPB Postop: 2 × 0.5 mg/day for 5 days	i.v.	Placebo
**15**	**Polymeropoulos 2015** [35]	22 cardiac surgery with CPB	Preop: 4x 500 mg/d for 2 days prior to surgery Postop: 4x 500 mg/d for 4 days	i.v.	Placebo
**16**	**Sadeghpour 2015** [19]	290 elective CABG or valve	Preop: 1 × 2 g immediately before surgery Postop: 1 × 1 g/day for 4 days	Preop: i.v. Postop: p.o.	Placebo
**17**	**Safaei 2017** [36]	87 elective isolated CABG with CPB	Group 1: 4 × 100 mg GSE 24 h before operation Group 2: 25 mg/kg vitamin C during surgery	i.v.	Standard of care
**18**	**Sarzaeem 2014** [37]	170 CABG	Preop: 2 g the night before surgery Postop: 2 × 500 mg/day for 5 days	i.v.	Placebo
**19**	**Van Wagoner 2003** [23]	346 CABG	Preop: 2 g the night before surgery Postop: 2 × 500 mg/day for 5 days	n.a.	Placebo

## Supplementary Materials

The following are available online at https://www.mdpi.com/2072-6643/11/9/2103/s1, File S1: Risk of bias assessment.

## Abbreviations

CABG	Coronary artery bypass graft
CPB	Cardio pulmonary bypass
CI	Confidence interval
GSE	Grape seed extract
ICU	Intensive Care Unit
LOS	Length-of-stay
n.a.	Not available
p.o.	Per os/orally
Postop	After surgery
Preop	Before surgery
RCT	Randomized controlled trial
Vit C	Vitamin C

## Appendix A. Manuscripts Excluded from This Analysis After Full Text Screening

	**Author and Year**	**Rationale for Exclusion**
1	Ali-Hasan Al Saegh 2016 [42]	Systematic review and meta-analysis
2	Ali-Hassan-Sayegh 2014 [40]	Systematic review and meta-analysis
3	Baines 2002 [43]	Review
4	Baker 2016 [11]	Meta-analysis
5	Carnes 2001 [44]	No randomization and retrospective control group
6	Das 2016 [45]	Use of inappropriate medication in control group (antacid instead of placebo)
7	Dingchao 1994 [46]	No randomization, „divided into two groups”
8	Ebade 2014 [47]	No randomization, „divided into three equal groups”
9	Hemilae 2017 [13]	Systematic review and meta-analysis
10	Hill 2018 [4]	Review
11	Hu 2017 [14]	Meta-analysis
12	Kumar 2013 [48]	Meta-analysis
13	Li 1990 [49]	Randomization strategy not mentioned in abstract. Full text not accessible, attempts to contact author unsuccessful.
14	Liu 2010 [50]	Letter to the editor
15	Moludi 2016 [51]	Paper and abstract within the university´s journal, abstract identical to included publiction by Sadeghpour et al. [19]
16	Oktar 2001 [52]	No randomization
17	Oudemans-van Straten [7]	Review
18	Polymeropoulos 2016 [12]	Meta-analysis
19	Rasoli 2011 [53]	Review
20	Rodrigo 2008 [54]	Review
21	Rodrigo 2009 [55]	Review
22	Rebrova [56]	No randomization
23	Sisto 1995 [57]	Inappropriate comedication (allopurinol)
24	Samadikah 2014 [58]	Inappropriate comedication (statin)
25	Shi 2018 [15]	Meta-analysis
26	NCT00519337	Registered trial on clinicaltrials.gov, no results posted or found
28	NCT01167569	Registered trial on clinicaltrials.gov. Contact to author unsuccessful, no results posted or found

## Appendix B. Methods for Risk of Bias Assessment

### Appendix B.1. Selection BIAS

For each included study, we described the method used to generate the allocation sequence in sufficient detail to allow an assessment of whether it should produce comparable groups. The method was assessed as follows:

- Low risk (any truly random process, e.g., random number table; computer random number generator);
- High risk (any non-random process, e.g., odd or even date of birth; hospital or clinic record number);
- Unclear risk (insufficient information to permit judgement).

### Appendix B.2. Allocation Concealment

For each included study, we described the method used to conceal the allocation sequence and determine whether the intervention allocation could have been foreseen in advance of or during recruitment, or changed after assignment. We assessed the method as follows:

- Low risk (e.g., telephone or central randomization; consecutively numbered, sealed, opaque envelopes);
- High risk (open random allocation; unsealed or non-opaque envelopes; alternation; date of birth);
- Unclear risk (insufficient information to permit judgement).

### Appendix B.3. Performance Bias

We described whether participants and personnel were blind to the allocation to the intervention or control groups in our ’Risk of bias’ assessment. We assessed the methods as:

- Low, high or unclear risk of bias for participants;
- Low, high or unclear risk of bias for personnel.

### Appendix B.4. Detection Bias

For each included study, we described the methods used, if any, to blind outcome assessors from knowledge of which intervention a participant received. We considered studies to be at low risk of bias if they were blinded or if we judged that the lack of blinding could not have affected the results. We assessed the method as follows:

- Low risk (no blinding of outcome assessment but the authors judged that the outcome was not likely to be influenced by this);
- High risk (no blinding of outcome assessment and the outcome measurement was likely to have been influenced by this);
- Unclear risk (insufficient information to permit judgement; the study did not address this).

### Appendix B.5. Attrition Bias

For each included study and for each outcome or class of outcomes, we described the completeness of data including attrition and exclusions from the analysis. We state whether attrition and exclusions were reported, the numbers included in the analysis at each stage (compared with the total number of randomized participants), reasons for attrition or exclusion where reported, and whether missing data are balanced across groups or are related to outcomes. We assessed the method as follows:

- Low risk (20% or less missing data);
- High risk (more than 20% missing data);
- Unclear risk (insufficient reporting to permit judgement; the study did not address this).

### Appendix B.6. Reporting Bias

Selective reporting (checking for reporting bias): We investigated the possibility of selective outcome reporting bias by identifying the outcomes in the study protocol (if available) and in the methods section of the publication, and by crosschecking to see if these outcomes were reported in the results section of the trial publication(s). We assessed the method as follows:

- Low risk (where it was clear that all of the study’s prespecified outcomes as identified in the study protocol (where available) and in the methods section were reported on; that all expected outcomes of interest to the review were reported on);
- High risk (where it was clear that not all of the study’s prespecified outcomes as identified in the study protocol (where available) and in the methods section were reported on; failure to include a key outcome that would have been expected to have been included);
- Unclear risk (insufficient information to permit judgement).

### Appendix B.7. Other Bias

For each included study, we described any important concerns we had about other possible sources of bias, for example sources of research funding. We assessed whether each study was free of other problems that could put it at risk of bias as follows:

- Low risk (study appeared to be free of bias);
- High risk (had at least one important risk of bias, for example related to study design);
- Unclear risk (insufficient information to permit judgement).
