# Correction: Hill, A.; et al. Effects of Vitamin C on Organ Function in Cardiac Surgery Patients: A Systematic Review and Meta-Analysis. *Nutrients* 2019, *11*, 2103

**DOI:** 10.3390/nu12123910

**Published:** 2020-12-21

**Authors:** Aileen Hill, Kai C. Clasen, Sebastian Wendt, Ádám G. Majoros, Christian Stoppe, Neill KJ Adhikari, Daren K. Heyland, Carina Benstoem

**Affiliations:** 1 Department of Intensive Care Medicine, Medical Faculty RWTH Aachen, D-52074 Aachen, Germany; kaclasen@ukaachen.de (K.C.C.); adam.majoros@rwth-aachen.de (Á.G.M.); christian.stoppe@googlemail.com (C.S.); 2 Department of Anesthesiology, Medical Faculty RWTH Aachen, D-52074 Aachen, Germany; wendt_s@gmx.net; 3 3CARE—Cardiovascular Critical Care and Anesthesia Evaluation and Research, D-52074 Aachen, Germany; 4 Medical Faculty, RWTH Aachen University, D-52074 Aachen, Germany; 5 Department of Critical Care Medicine, Sunnybrook Health Sciences Centre, Interdepartmental Division of Critical Care Medicine, University of Toronto, Toronto, ON M4N 3M5, Canada; neill.adhikari@utoronto.ca; 6 Clinical Evaluation Research Unit, Kingston General Hospital, Kingston, CA K7L 2V7, Canada; dkh2@queensu.ca

The authors thank the readers for pointing out the issues [1] in their publication [2], and wish to make a correction in the published version of their paper as the response of the readers’ Comment. *p*-values have been corrected in the following parts of the paper.

In Abstract: Vitamin C significantly decreased the incidence of atrial fibrillation (*p* < 0.0001), ventilation time (*p* = 0.0003), intensive care unit (ICU) length-of-stay (*p* = 0.002), and hospital length of stay (*p* = 0.03). However, on average, vitamin C had no significant effects on in hospital mortality (*p* = 0.59), or on the incidence of stroke (*p* = 0.32).

In Section 4.2. Cardiac Function

On average, a significant effect in favor of vitamin C was observed (*p* < 0.0001, CI 0.46 to 0.77).

In Section 4.3. Pulmonary Function

On average, the effect of vitamin C was significant on reduction of ventilation time (*p* = 0.0003, CI −3.99 to −1.18); we observed no statistical heterogeneity (I^2^ = 0%).

In Section 4.6. In-Hospital Mortality

On average, no significant effect of vitamin C was found on in-hospital mortality (*p* = 0.59, CI 0.21 to 2.40).

In Section 4.7. Length of Stay

On average, a significant effect favoring vitamin C administration regarding ICU length-of stay (LOS) was detected (*p* = 0.002, CI −9.66 to −2.14).

On average, there was a significant effect in favor of vitamin C regarding hospital LOS (*p* = 0.03, CI −34.49 to −1.41).

In Section 4.8. Subgroup Analysis Influence of Administration Route: Intravenous Administration versus Oral Administration of Vitamin C.

A total of eight studies contributed reported the outcome “cerebral ischemic events” and contributed to the subgroup analysis investigating any possible influence of the route of administration. In four of the eight studies, cerebral ischemic events occurred and the evidence suggested no difference in treatment effect (test for subgroup differences (Chi^2^ = 0.09, *df* = 1 (*p* = 0.99), I^2^ = 0%).

A total of eight studies contributed to the subgroup analysis investigating any possible influence of the route of administration on the outcome incidence of “atrial fibrillation”. While the effect of the treatment was statistically significant in the group receiving intravenous vitamin C (*p* = 0.002, CI 0.53 to 0.87, I^2^ = 0%), it was not in patients receiving oral vitamin C (*p* = 0.09, CI 0.19 to 1.13, I^2^ = 74%). However, the test for subgroup differences (Chi^2^ = 0.69, *df* = 1 (*P* = 0.41), I^2^ = 0%) shows no evidence that the effect of vitamin C is different between the trials that used intravenous administration and those that used oral administration on the outcome incidence of “atrial fibrillation”.

A total of four studies contributed to the subgroup analysis investigating any possible influence of the route of administration on the outcome “duration of mechanical ventilation”. We found a statistical significance in the group receiving intravenous vitamin C (*p* = 0.05, CI −15.52 to −0.08, I^2^ not applicable); however, this group included only one RCT with 58 patients in total. In the group of oral vitamin C administration, the treatment effect did not reach statistical significance (*p* = 0.10, CI −6.22 to 0.54, I^2^ = 0%). The test for subgroup differences (Chi^2^ = 1.89, *df* = 2 (*p* = 0.39), I^2^ = 0%) shows no evidence that the effect of vitamin C is different between the trials that used intravenous administration and those that used oral administration on the outcome incidence of “duration of mechanical ventilation”.

A total of eight studies contributed to the subgroup analysis investigating any possible influence of the route of administration on the outcome “in-hospital mortality”. We found no evidence of a treatment effect between subgroups (test for subgroup differences (Chi^2^ = 0.26, *df* = 1 (*p* = 0.61), I^2^ = 0%).

A total of nine studies contributed to the subgroup analysis investigating any possible influence of the route of administration on the outcome “ICU-LOS”. We found no statistically significant effects in the group receiving intravenous vitamin C (*p* = 0.12, CI −9.64 to 1.07, I^2^ = 68%), but in the group of oral vitamin C administration, the treatment effect did reach statistical significance (*p* = 0.0003, CI −11.98 to −3.53, I^2^ = 0%). The test for subgroup differences (Chi^2^ = 1.00, *df* = 1 (*p* = 0.32), I^2^ = 0%) shows no evidence that the effect of vitamin C is different between the trials that used intravenous administration and those that used oral administration on the outcome “ICU-LOS”.

A total of eight studies contributed to the subgroup analysis investigating any possible influence of the route of administration on the outcome “hospital-LOS”. We found no statistical significance in the group receiving intravenous vitamin C (*p* = 0.36, CI −45.51 to 16.71, I^2^ = 91%), but in the group of oral vitamin C administration, the treatment effect did reach statistical significance (*p* = 0.01, CI −20.07 to −2.36, I^2^ = 81%). The test for subgroup differences (Chi^2^ = 0.04, *df* = 1 (*p* = 0.85), I^2^ = 0%) shows no evidence that the effect of vitamin C is different between the trials that used intravenous administration and those that used oral administration on the outcome “hospital-LOS”.

In Section 4.9. Subgroup Analysis Influence of Control Group: “Vitamin C versus Placebo” versus “Vitamin C versus Standard of Care”.

A total of eight studies reported the outcome “cerebral ischemic events” and contributed to the subgroup analysis investigating any possible influence of the control group on the outcome. In four of the eight studies, cerebral ischemic events occurred. We found no evidence of a treatment effect between subgroups (test for subgroup differences (Chi^2^ = 0.55, *df* = 1 (*p* = 0.46), I^2^ = 0%).

A total of thirteen studies contributed to the subgroup analysis investigating any possible influence of the control group on the outcome incidence of “atrial fibrillation”. We found evidence of a treatment effect between subgroups (test for subgroup differences (Chi^2^ = 11.84, *df* = 1 (*p* = 0.0006), I^2^ = 91.6%) between the trials comparing placebo and trials comparing standard of care for the outcome “atrial fibrillation”.

A total of four studies contributed to the subgroup analysis investigating any possible influence of the control group on the outcome “duration of mechanical ventilation. We found a statistical significance in the group comparing vitamin C to placebo (*p* = 0.002, CI −3.99 to −0.93, I^2^ = 0%), but not in the group comparing vitamin C to standard of care (*p* = 0.15, CI −9.25 to 1.38, I^2^ = 41%). The test for subgroup differences (Chi^2^ = 0.27, *df* = 1 (*p* = 0.60), I^2^ = 0%) shows no evidence that the effect of vitamin C is different between the trials that with compared to placebo and those that compared to standard of care on the outcome “duration of mechanical ventilation”.

A total of nine studies reported the outcome “in-hospital mortality” and contributed to the subgroup analysis investigating any possible influence of the control group on the outcome. In-hospital deaths occurred in only four of these studies and we found no evidence of a treatment effect between subgroups (test for subgroup differences (Chi^2^ = 0.12, *df* = 1 (*p* = 0.73), I^2^ = 0%).

A total of 11 studies contributed to the subgroup analysis investigating any possible influence of the control group on the outcome “ICU-LOS”. We found no evidence of a treatment effect between subgroups (test for subgroup differences (Chi^2^ = 0.52, *df* = 1 (*p* = 0.47), I^2^ = 0%).

A total of eight studies contributed to the subgroup analysis investigating any possible influence of the control group on the outcome “hospital-LOS”. We found a statistical significance in the placebo group (*p* < 0.00001, CI −50.48 to −29.85, I^2^ = 0%). In the standard care group, the treatment effect did not reach statistical significance (*p* = 0.89, CI −13.90 to 16.10, I^2^ = 63%). The test for subgroup differences (Chi^2^ = 19.74, *df* = 1 (*p* < 0.00001), I^2^ = 94.9%) shows evidence that the effect of vitamin C is different between the trials compared to the placebo and those that compared to standard of care on the outcome “hospital-LOS”.

In Section 5. Discussion
In Section 5.1. Quality of the Evidence

The low total number of adverse events as well as the low number of studies contributing to some subgroup analyses (for example “cerebral ischemic events” and “duration of mechanical ventilation”) limit the conclusions that may be drawn from the meta-analyses.

In Section 5.3. Agreements and Disagreements with Other Reviews.

Three meta-analyses also found significantly shorter hospital-LOS associated with vitamin C.

The authors also add additional appendix materials as below.

## Supplementary Materials

The following are available online at https://www.mdpi.com/2072-6643/12/12/3910/s1, due to major changes and efforts for the revision of original manuscript (doi:10.3390/nu11092103), please see all details of revised paper in supplementary material

## Appendix C. Risk of Bias Assessment

**Table A1:** **Alshafey 2017** [3].

Bias	Authors’ Judgement	Support for Judgement
Random sequence generation (selection bias)	Unclear risk	Insufficient information to form judgement
Allocation concealment (selection bias)	Unclear risk	Insufficient information to form judgement
Blinding of participants and personnel (performance bias)	High risk	No blinding of participants and personnel
Blinding of outcome assessment (detection bias)	High risk	No blinding of outcome assessment
Incomplete outcome data (attrition bias)	Low risk	All data reported
Selective reporting (reporting bias)	Unclear risk	All outcomes stated in the methods section were adequately reported or explained in results
Other bias	Unclear risk	Funding for trial: not reported Notable conflicts of interest of authors: not reported

**Table A2:** **Antonic 2016** [4].

Bias	Authors’ Judgement	Support for Judgement
Random sequence generation (selection bias)	Unclear risk	Insufficient information to form judgement
Allocation concealment (selection bias)	Unclear risk	Insufficient information to form judgement
Blinding of participants and personnel (performance bias)	High risk	No blinding of participants and personnel
Blinding of outcome assessment (detection bias)	High risk	No blinding of outcome assessment
Incomplete outcome data (attrition bias)	Low risk	All data reported
Selective reporting (reporting bias)	Low risk	All outcomes stated in the methods section were adequately reported or explained in results
Other bias	Low risk	Funding for trial: no funding Notable conflicts of interest of authors: all authors declare no conflict of interest

**Table A3:** **Antonic 2017** [5].

Bias	Authors’ Judgement	Support for Judgement
Random sequence generation (selection bias)	Unclear risk	Insufficient information to form judgement
Allocation concealment (selection bias)	Unclear risk	Insufficient information to form judgement
Blinding of participants and personnel (performance bias)	High risk	No blinding of participants and personnel
Blinding of outcome assessment (detection bias)	High risk	No blinding of outcome assessment
Incomplete outcome data (attrition bias)	Low risk	All data reported
Selective reporting (reporting bias)	Low risk	All outcomes stated in the methods section were adequately reported or explained in results
Other bias	Unclear risk	Funding for trial: not reported Notable conflicts of interest of authors: not reported

**Table A4:** **Bakr 2015** [6].

Bias	Authors’ Judgement	Support for Judgement
Random sequence generation (selection bias)	Unclear risk	Insufficient information to form judgement
Allocation concealment (selection bias)	Unclear risk	Insufficient information to form judgement
Blinding of participants and personnel (performance bias)	Unclear risk	Insufficient information to form judgement
Blinding of outcome assessment (detection bias)	Unclear risk	Insufficient information to form judgement
Incomplete outcome data (attrition bias)	Unclear risk	Insufficient information to form judgement
Selective reporting (reporting bias)	Unclear risk	Insufficient information to form judgement
Other bias	Unclear risk	Funding for trial: not reported Notable conflicts of interest of authors: not reported

**Table A5:** **Bjordahl 2012** [7].

Bias	Authors’ Judgement	Support for Judgement
Random sequence generation (selection bias)	Unclear risk	Insufficient information to form judgement
Allocation concealment (selection bias)	Low risk	“The pharmacy department maintained the randomization list andassigned participants to the [...] arms of the study in a blinded fashion.”
Blinding of participants and personnel (performance bias)	Low risk	“Participants, clinicians, and evaluators were blinded to the treatment assignments and the blind was not broken until after data analyses were complete.”
Blinding of outcome assessment (detection bias)	Low risk	“[…] evaluators were blinded to the treatment assignments and the blind was not broken until after data analyses were complete”
Incomplete outcome data (attrition bias)	Low risk	All data reported
Selective reporting (reporting bias)	Low risk	All outcomes stated in the methods section were adequately reported or explained in results
Other bias	Low risk	Funding for trial: not reported Notable conflicts of interest of authors: all authors report no conflict of interest

**Table A6:** **Colby 2011** [8].

Bias	Authors’ Judgement	Support for Judgement
Random sequence generation (selection bias)	Low risk	“Eligible patients were randomized using a computer-generated sequence with a 1:1 allocation and a random block size of 10.”
Allocation concealment (selection bias)	Low risk	“Eligible patients were randomized using a computer-generated sequence with a 1:1 allocation and a random block size of 10.”
Blinding of participants and personnel (performance bias)	Low risk	“Study patients, cardiothoracic surgeons, caregivers, and investigators, including those responsible for data collection, were blinded to the treatment allocation.”
Blinding of outcome assessment (detection bias)	Low risk	“Study patients, cardiothoracic surgeons, caregivers, and investigators, including those responsible for data collection, were blinded to the treatment allocation.”
Incomplete outcome data (attrition bias)	Low risk	All data reported (one patient excluded from analysis as the patient did not receive the study drug)
Selective reporting (reporting bias)	Low risk	All outcomes stated in the methods section were adequately reported or explained in results
Other bias	Low risk	Funding for trial: Gustavus and Luise Pfeiffer Research Foundation, the sponsor played no role in the design, execution, analysis or submission of the trial and its results Notable conflicts of interest of authors: all authors report no conflict of interest

**Table A7:** **Dehghani 2014** [9].

Bias	Authors’ Judgement	Support for Judgement
Random sequence generation (selection bias)	Low risk	“Patients were randomized into two groups in a 1:1 ratio using random-number table.”
Allocation concealment (selection bias)	Unclear risk	Insufficient information to form judgement
Blinding of participants and personnel (performance bias)	High risk	No blinding of participants and personnel
Blinding of outcome assessment (detection bias)	High risk	No blinding of outcome assessment
Incomplete outcome data (attrition bias)	Low risk	All data reported
Selective reporting (reporting bias)	Low risk	All outcomes stated in the methods section were adequately reported or explained in results
Other bias	Unclear risk	Funding for trial: not reported Notable conflicts of interest of authors: all authors report no conflict of interest

**Table A8:** **Demirag 2001** [10].

Bias	Authors’ Judgement	Support for Judgement
Random sequence generation (selection bias)	Unclear risk	Insufficient information to form judgement
Allocation concealment (selection bias)	Unclear risk	Insufficient information to form judgement
Blinding of participants and personnel (performance bias)	High risk	No blinding of participants and personnel
Blinding of outcome assessment (detection bias)	High risk	No blinding of outcome assessment
Incomplete outcome data (attrition bias)	Low risk	All data reported
Selective reporting (reporting bias)	Low risk	All outcomes stated in the methods section were adequately reported or explained in results
Other bias	Unclear risk	Funding for trial: not reported Notable conflicts of interest of authors: not reported

**Table A9:** **Donovan 2012** [11].

Bias	Authors’ Judgement	Support for Judgement
Random sequence generation (selection bias)	Unclear risk	Insufficient information to form judgement
Allocation concealment (selection bias)	Unclear risk	Insufficient information to form judgement
Blinding of participants and personnel (performance bias)	Unclear risk	Insufficient information to form judgement
Blinding of outcome assessment (detection bias)	Unclear risk	Insufficient information to form judgement
Incomplete outcome data (attrition bias)	Unclear risk	Insufficient information to form judgement
Selective reporting (reporting bias)	Unclear risk	Insufficient information to form judgement
Other bias	Unclear risk	Funding for trial: not reported Notable conflicts of interest of authors: not reported

**Table A10:** **Eslami 2007** [12].

Bias	Authors’ Judgement	Support for Judgement
Random sequence generation (selection bias)	Unclear risk	Insufficient information to form judgement
Allocation concealment (selection bias)	Unclear risk	Insufficient information to form judgement
Blinding of participants and personnel (performance bias)	High risk	No blinding of participants and personnel
Blinding of outcome assessment (detection bias)	Low risk	“Echocardiography [..] was performed before surgery by a single investigator in a blinded fashion.” “All of the Holter recordings were examined by a single investigator who had been blinded to patients’ group assignments.”
Incomplete outcome data (attrition bias)	Low risk	All data reported
Selective reporting (reporting bias)	Low risk	All outcomes stated in the methods section were adequately reported or explained in results
Other bias	Unclear risk	Funding for trial: This study was supported in part by a research grant from Tehran University of Medical SciencesNotable conflicts of interest of authors: not reported

**Table A11:** **Healy 2010** [13].

Bias	Authors’ Judgement	Support for Judgement
Random sequence generation (selection bias)	Unclear risk	Insufficient information to form judgement
Allocation concealment (selection bias)	Unclear risk	Insufficient information to form judgement
Blinding of participants and personnel (performance bias)	High risk	No blinding of participants and personnel
Blinding of outcome assessment (detection bias)	Unclear risk	Insufficient information to form judgement
Incomplete outcome data (attrition bias)	High risk	Interim analysis of only 60 patients reported as abstract only
Selective reporting (reporting bias)	Unclear risk	Insufficient information to form judgement
Other bias	Unclear risk	Funding for trial: not reported Notable conflicts of interest of authors: not reported

**Table A12:** **Jouybar 2012** [14].

Bias	Authors’ Judgement	Support for Judgement
Random sequence generation (selection bias)	Low risk	“The patients were randomly assigned to two groups according to the printed table of random numbers, to either receive [...].” “A blinded anesthesiologist who was involved neither in the patients’ allocation and management nor in the design of the study and data processing and analysis, generated the randomization list using a computer program.”
Allocation concealment (selection bias)	Unclear risk	Insufficient information to form judgement
Blinding of participants and personnel (performance bias)	Unclear risk	“Moreover, the physician responsible for managing the patients did not participate in the study.”
Blinding of outcome assessment (detection bias)	Low risk	No blinding of outcome assessment, however, only outcomes were laboratory measures, lack of blinding has minor impact of evaluation of these endpoints
Incomplete outcome data (attrition bias)	Low risk	10% of patients not treated according to protocol, excluded from analysis
Selective reporting (reporting bias)	Unclear risk	All outcomes stated in the methods section were adequately reported or explained in results
Other bias	Low risk	Funding for trial: This work was supported by Shiraz University of Medical Sciences Notable conflicts of interest of authors: all authors report no conflict of interest

**Table A13:** **Knodell 1981** [15].

Bias	Authors’ Judgement	Support for Judgement
Random sequence generation (selection bias)	Unclear risk	Insufficient information to form judgement
Allocation concealment (selection bias)	Unclear risk	Insufficient information to form judgement
Blinding of participants and personnel (performance bias)	Low risk	“double-blind trial”
Blinding of outcome assessment (detection bias)	Low risk	“Clinical and laboratory data sheets on all patients with even minor enzyme elevations were submitted to two independent rereviewers [...] for evaluation.” “These reviewers either accepted or rejected patients as cases of posttransfusion hepatitis, and analysis of data was based on their decisions.”
Incomplete outcome data (attrition bias)	High risk	“40 patients [...] who did not complete the study were distributed equally between the placebo and vitamin C treatment groups. The vast majority of patients who did not complete the study either refused to take the study medication postoperatively (11 patients in each group) or refused to have follow-up blood samples drawn.”
Selective reporting (reporting bias)	High risk	All outcomes stated in the methods section are NOT adequately reported or explained in results: Serum aminotransferases (only SGPT, SGOT missing Alkaline phosphatase missing Symptoms of congestive heart failure, one month intervals missing
Other bias	Unclear risk	Funding for trial: Hoffmann-LaRoche and the Veterans Research Service Notable conflicts of interest of authors: not reported

**Table A14:** **Papoulidis 2011** [16].

Bias	Authors’ Judgement	Support for Judgement
Random sequence generation (selection bias)	High risk	“The initial random assignment was by flipping a coin but simple randomization led to an imbalance with respect to sample size with a treatment group of 130 patients and control group of 85 patients. In order to have an equal sample size, we reevaluated our randomization protocol and using a random generator, the computer chose 85 out of 130 patients which were initially enrolled in the study group.”
Allocation concealment (selection bias)	Low risk	“The initial random assignment was by flipping a coin but simple randomization led to an imbalance with respect to sample size with a treatment group of 130 patients and control group of 85 patients. In order to have an equal sample size, we reevaluated our randomization protocol and using a random generator, the computer chose 85 out of 130 patients which were initially enrolled in the study group.”
Blinding of participants and personnel (performance bias)	High risk	No blinding of participants and personnel
Blinding of outcome assessment (detection bias)	Low risk	“Echocardiography was performed before surgery by a single echocardiographer in a blinded fashion.”
Incomplete outcome data (attrition bias)	Low risk	All data reported
Selective reporting (reporting bias)	Low risk	All outcomes stated in the methods section were adequately reported or explained in results
Other bias	Unclear risk	Funding for trial: not reported Notable conflicts of interest of authors: not reported

**Table A15:** **Polymeropoulos 2015** [17].

Bias	Authors’ Judgement	Support for Judgement
Random sequence generation (selection bias)	Unclear risk	Insufficient information to form judgement
Allocation concealment (selection bias)	Unclear risk	Insufficient information to form judgement
Blinding of participants and personnel (performance bias)	Unclear risk	Insufficient information to form judgement
Blinding of outcome assessment (detection bias)	Unclear risk	Insufficient information to form judgement
Incomplete outcome data (attrition bias)	Low risk	All data reported
Selective reporting (reporting bias)	Unclear risk	Insufficient information to form judgement
Other bias	Unclear risk	Funding for trial: not reported Notable conflicts of interest of authors: not reported

**Table A16:** **Sadeghpour 2015** [18].

Bias	Authors’ Judgement	Support for Judgement
Random sequence generation (selection bias)	Low risk	“The study population was randomized one day before surgery to two groups (by using www.randomaizer.org). The method of randomization was balanced block with an allocation sequence based on a block size of eight, generated with a computer random number generator.”
Allocation concealment (selection bias)	Low risk	“The study population was randomized one day before surgery to two groups (by using www.randomaizer.org). The method of randomization was balanced block with an allocation sequence based on a block size of eight, generated with a computer random number generator.”
Blinding of participants and personnel (performance bias)	Low risk	“Both the patients and the hospital staff were blind to the treatment allocation.”
Blinding of outcome assessment (detection bias)	Unclear risk	Insufficient information to form judgement
Incomplete outcome data (attrition bias)	Low risk	All data reported
Selective reporting (reporting bias)	Low risk	All outcomes stated in the methods section were adequately reported or explained in results
Other bias	Unclear risk	Funding for trial: not reported Notable conflicts of interest of authors: not reported

**Table 17:** **Safaei 2017** [19].

Bias	Authors’ Judgement	Support for Judgement
Random sequence generation (selection bias)	Low risk	“Patients were randomly assigned to three groups (*n* = 29 each) using random allocation software.”
Allocation concealment (selection bias)	Low risk	“Patients were randomly assigned to three groups (*n* = 29 each) using random allocation software.”
Blinding of participants and personnel (performance bias)	High risk	No blinding of participants and personnel.
Blinding of outcome assessment (detection bias)	Low risk	“All data were collected by an independent research nurse assigned to this research study and were blinded to the groups.”“All clinical data were collected by an independent end-point assessor team including a cardiologist and a nurse who were assigned to this clinical trial and were blinded to group assignment.”
Incomplete outcome data (attrition bias)	Low risk	Less than 20% lost to follow-up
Selective reporting (reporting bias)	Low risk	All outcomes stated in the methods section were adequately reported or explained in results
Other bias	Unclear risk	Funding for trial: Cardiovascular Research Center, Tabriz University of Medical Sciences, Tabriz, Iran Notable conflicts of interest of authors: all authors report no conflict of interest

**Table A18:** **Sarzaeem 2014** [20].

Bias	Authors’ Judgement	Support for Judgement
Random sequence generation (selection bias)	Unclear risk	Insufficient information to form judgement, study reported in Farsi, translation difficult
Allocation concealment (selection bias)	Unclear risk	Insufficient information to form judgement, study reported in Farsi, translation difficult
Blinding of participants and personnel (performance bias)	Unclear risk	Insufficient information to form judgement, study reported in Farsi, translation difficult
Blinding of outcome assessment (detection bias)	Unclear risk	Insufficient information to form judgement, study reported in Farsi, translation difficult
Incomplete outcome data (attrition bias)	Unclear risk	Insufficient information to form judgement, study reported in Farsi, translation difficult
Selective reporting (reporting bias)	Unclear risk	Insufficient information to form judgement, study reported in Farsi, translation difficult
Other bias	Unclear risk	Insufficient information to form judgement, study reported in Farsi, translation difficult

**Table A19:** **Van Wagoner 2003** [21].

Bias	Authors’ Judgement	Support for Judgement
Random sequence generation (selection bias)	Unclear risk	Insufficient information to form judgement
Allocation concealment (selection bias)	Unclear risk	Insufficient information to form judgement
Blinding of participants and personnel (performance bias)	Unclear risk	Insufficient information to form judgement
Blinding of outcome assessment (detection bias)	Unclear risk	Insufficient information to form judgement
Incomplete outcome data (attrition bias)	Unclear risk	Insufficient information to form judgement
Selective reporting (reporting bias)	Unclear risk	Insufficient information to form judgement
Other bias	Unclear risk	Funding for trial: not reported Notable conflicts of interest of authors: all authors report no conflict of interest
