# Supplementary material for: Effects of Vitamin C on Organ Function in Cardiac Surgery Patients: A Systematic Review and Meta-Analysis
Source: Nutrients. 2019 Sep 4;11(9):2103. doi: 10.3390/nu11092103 (PMC6769534; doi:10.3390/nu11092103)
Supplement: Supplementary file 1 [file nutrients-11-02103-s001.pdf]

Supplementary Materials: Risk of bias assessment

Alshafey 2017

**Bias**	**Authors' judgement**	**Support for judgement**
Random sequence generation (selection bias)	Unclear risk	Insufficient information to form judgement
Allocation concealment (selection bias)	Unclear risk	Insufficient information to form judgement
Blinding of participants and personnel (performance bias)	High risk	No blinding of participants and personnel
Blinding of outcome assessment (detection bias)	High risk	No blinding of outcome assessment
Incomplete outcome data (attrition bias)	Low risk	All data reported
Selective reporting (reporting bias)	Unclear risk	All outcomes stated in the methods section were adequately reported or explained in results
Other bias	Unclear risk	Funding for trial: not reported<br>Notable conflicts of interest of authors: not reported

Antonic 2016

Bias	Authors' judgement	Support for judgement
Random sequence generation (selection bias)	Unclear risk	Insufficient information to form judgement
Allocation concealment (selection bias)	Unclear risk	Insufficient information to form judgement
Blinding of participants and personnel (performance bias)	High risk	No blinding of participants and personnel
Blinding of outcome assessment (detection bias)	High risk	No blinding of outcome assessment
Incomplete outcome data (attrition bias)	Low risk	All data reported
Selective reporting (reporting bias)	Low risk	All outcomes stated in the methods section were adequately reported or explained in results
Other bias	Low risk	Funding for trial: no funding<br>Notable conflicts of interest of authors: all authors declare no conflict of interest

Antonic 2017

Bias	Authors' judgement	Support for judgement
Random sequence generation (selection bias)	Unclear risk	Insufficient information to form judgement
Allocation concealment (selection bias)	Unclear risk	Insufficient information to form judgement
Blinding of participants and personnel (performance bias)	High risk	No blinding of participants and personnel
Blinding of outcome assessment (detection bias)	High risk	No blinding of outcome assessment
Incomplete outcome data (attrition bias)	Low risk	All data reported
Selective reporting (reporting bias)	Low risk	All outcomes stated in the methods section were adequately reported or explained in results
Other bias	Unclear risk	Funding for trial: not reported<br>Notable conflicts of interest of authors: not reported

Bakr 2015

Bias	Authors' judgement	Support for judgement
Random sequence generation (selection bias)	Unclear risk	Insufficient information to form judgement
Allocation concealment (selection bias)	Unclear risk	Insufficient information to form judgement
Blinding of participants and personnel (performance bias)	Unclear risk	Insufficient information to form judgement
Blinding of outcome assessment (detection bias)	Unclear risk	Insufficient information to form judgement
Incomplete outcome data (attrition bias)	Unclear risk	Insufficient information to form judgement
Selective reporting (reporting bias)	Unclear risk	Insufficient information to form judgement
Other bias	Unclear risk	Funding for trial: not reported<br>Notable conflicts of interest of authors: not reported

Bjordahl 2012

Bias	Authors' judgement	Support for judgement
Random sequence generation (selection bias)	Unclear risk	Insufficient information to form judgement
Allocation concealment (selection bias)	Low risk	"The pharmacy department maintained the randomization list and<br>assigned participants to the [...] arms of the study in a blinded fashion."
Blinding of participants and personnel (performance bias)	Low risk	"Participants, clinicians, and evaluators were blinded to the treatment assignments and the blind was not broken until after data analyses were complete."
Blinding of outcome assessment (detection bias)	Low risk	"[…] evaluators were blinded to the treatment assignments and the blind was not broken until after data analyses were complete"
Incomplete outcome data (attrition bias)	Low risk	All data reported
Selective reporting (reporting bias)	Low risk	All outcomes stated in the methods section were adequately reported or explained in results
Other bias	Low risk	Funding for trial: not reported<br>Notable conflicts of interest of authors: all authors report no conflict of interest

Colby 2011

**Bias**	**Authors' judgement**	**Support for judgement**
Random sequence generation (selection bias)	Low risk	"Eligible patients were randomized using a computer-generated sequence with a 1:1 allocation and a random block size of 10."
Allocation concealment (selection bias)	Low risk	"Eligible patients were randomized using a computer-generated sequence with a 1:1 allocation and a random block size of 10."
Blinding of participants and personnel (performance bias)	Low risk	"Study patients, cardiothoracic surgeons, caregivers, and investigators, including those responsible for data collection, were blinded to the treatment allocation."
Blinding of outcome assessment (detection bias)	Low risk	"Study patients, cardiothoracic surgeons, caregivers, and investigators, including those responsible for data collection, were blinded to the treatment allocation."
Incomplete outcome data (attrition bias)	Low risk	All data reported (one patient excluded from analysis as the patient did not receive the study drug)
Selective reporting (reporting bias)	Low risk	All outcomes stated in the methods section were adequately reported or explained in results
Other bias	Low risk	Funding for trial: Gustavus and Luise Pfeiffer Research Foundation, the sponsor played no role in the design, execution, analysis or submission of the trial and its results<br>Notable conflicts of interest of authors: all authors report no conflict of interest

Dehghani 2014

Bias	Authors' judgement	Support for judgement
Random sequence generation (selection bias)	Low risk	"Patients were randomized into two groups in a 1:1 ratio using random-number table."
Allocation concealment (selection bias)	Unclear risk	Insufficient information to form judgement
Blinding of participants and personnel (performance bias)	High risk	No blinding of participants and personnel
Blinding of outcome assessment (detection bias)	High risk	No blinding of outcome assessment
Incomplete outcome data (attrition bias)	Low risk	All data reported
Selective reporting (reporting bias)	Low risk	All outcomes stated in the methods section were adequately reported or explained in results
Other bias	Unclear risk	Funding for trial: not reported<br>Notable conflicts of interest of authors: all authors report no conflict of interest

Demirag 2001

Bias	Authors' judgement	Support for judgement
Random sequence generation (selection bias)	Unclear risk	Insufficient information to form judgement
Allocation concealment (selection bias)	Unclear risk	Insufficient information to form judgement
Blinding of participants and personnel (performance bias)	High risk	No blinding of participants and personnel
Blinding of outcome assessment (detection bias)	High risk	No blinding of outcome assessment
Incomplete outcome data (attrition bias)	Low risk	All data reported
Selective reporting (reporting bias)	Low risk	All outcomes stated in the methods section were adequately reported or explained in results
Other bias	Unclear risk	Funding for trial: not reported<br>Notable conflicts of interest of authors: not reported

Donovan 2012

**Bias**	**Authors' judgement**	**Support for judgement**
Random sequence generation (selection bias)	Unclear risk	Insufficient information to form judgement
Allocation concealment (selection bias)	Unclear risk	Insufficient information to form judgement
Blinding of participants and personnel (performance bias)	Unclear risk	Insufficient information to form judgement
Blinding of outcome assessment (detection bias)	Unclear risk	Insufficient information to form judgement
Incomplete outcome data (attrition bias)	Unclear risk	Insufficient information to form judgement
Selective reporting (reporting bias)	Unclear risk	Insufficient information to form judgement
Other bias	Unclear risk	Funding for trial: not reported<br>Notable conflicts of interest of authors: not reported

Eslami 2007

**Bias**	**Authors' judgement**	**Support for judgement**
Random sequence generation (selection bias)	Unclear risk	Insufficient information to form judgement
Allocation concealment (selection bias)	Unclear risk	Insufficient information to form judgement
Blinding of participants and personnel (performance bias)	High risk	No blinding of participants and personnel
Blinding of outcome assessment (detection bias)	Low risk	"Echocardiography [..] was performed before surgery by a single investigator in a blinded fashion."<br>"All of the Holter recordings were examined by a single investigator who had been blinded to patients' group assignments."
Incomplete outcome data (attrition bias)	Low risk	All data reported
Selective reporting (reporting bias)	Low risk	All outcomes stated in the methods section were adequately reported or explained in results
Other bias	Unclear risk	Funding for trial: This study was supported in part by a research grant from Tehran University of Medical Sciences<br>Notable conflicts of interest of authors: not reported

Healy 2010

**Bias**	**Authors' judgement**	**Support for judgement**
Random sequence generation (selection bias)	Unclear risk	Insufficient information to form judgement
Allocation concealment (selection bias)	Unclear risk	Insufficient information to form judgement
Blinding of participants and personnel (performance bias)	High risk	No blinding of participants and personnel
Blinding of outcome assessment (detection bias)	Unclear risk	Insufficient information to form judgement
Incomplete outcome data (attrition bias)	High risk	Interim analysis of only 60 patients reported as abstract only
Selective reporting (reporting bias)	Unclear risk	Insufficient information to form judgement
Other bias	Unclear risk	Funding for trial: not reported<br>Notable conflicts of interest of authors: not reported

Jouybar 2012

Bias	Authors' judgement	Support for judgement
Random sequence generation (selection bias)	Low risk	"The patients were randomly assigned to two groups according to the printed table of random numbers, to either receive [...]."<br>"A blinded anesthesiologist who was involved neither in the patients' allocation and management nor in the design of the study and data processing and analysis, generated the randomization list using a computer program."
Allocation concealment (selection bias)	Unclear risk	Insufficient information to form judgement
Blinding of participants and personnel (performance bias)	Unclear risk	"Moreover, the physician responsible for managing the patients did not participate in the study."
Blinding of outcome assessment (detection bias)	Low risk	No blinding of outcome assessment, however, only outcomes were laboratory measures, lack of blinding has minor impact of evaluation of these endpoints
Incomplete outcome data (attrition bias)	Low risk	10% of patients not treated according to protocol, excluded from analysis
Selective reporting (reporting bias)	Unclear risk	All outcomes stated in the methods section were adequately reported or explained in results
Other bias	Low risk	Funding for trial: This work was supported by Shiraz University of Medical<br>Sciences<br>Notable conflicts of interest of authors: all authors report no conflict of interest

Knodell 1981

**Bias**	**Authors' judgement**	**Support for judgement**
Random sequence generation (selection bias)	Unclear risk	Insufficient information to form judgement
Allocation concealment (selection bias)	Unclear risk	Insufficient information to form judgement
Blinding of participants and personnel (performance bias)	Low risk	"double-blind trial"
Blinding of outcome assessment (detection bias)	Low risk	"Clinical and laboratory data sheets on all patients with even minor enzyme elevations were submitted to two independent rereviewers [...] for evaluation." "These reviewers either accepted or rejected patients as cases of posttransfusion hepatitis, and analysis of data was based on their decisions."
Incomplete outcome data (attrition bias)	High risk	"40 patients [...] who did not complete the study were distributed equally between the placebo and vitamin C treatment groups. The vast majority of patients who did not complete the study either refused to take the study medication postoperatively (11 patients in each group) or refused to have follow-up blood samples drawn."
Selective reporting (reporting bias)	High risk	All outcomes stated in the methods section are NOT adequately reported or explained in results:<br>Serum aminotransferases (only SGPT, SGOT missing<br>Alkaline phosphatase missing<br>Symptoms of congestive heart failure, one month intervals missing
Other bias	Unclear risk	Funding for trial: Hoffmann-LaRoche and the Veterans Research Service<br>Notable conflicts of interest of authors: not reported

Papoulidis 2011

Bias	Authors' judgement	Support for judgement
Random sequence generation (selection bias)	High risk	"The initial random assignment was by flipping a coin but simple randomization led to an imbalance with respect to sample size with a treatment group of 130 patients and control group of 85 patients. In order to have an equal sample size, we reevaluated our randomization protocol and using a random generator, the computer chose 85 out of 130 patients which were initially enrolled in the study group."
Allocation concealment (selection bias)	Low risk	"The initial random assignment was by flipping a coin but simple randomization led to an imbalance with respect to sample size with a treatment group of 130 patients and control group of 85 patients. In order to have an equal sample size, we reevaluated our randomization protocol and using a random generator, the computer chose 85 out of 130 patients which were initially enrolled in the study group."
Blinding of participants and personnel (performance bias)	High risk	No blinding of participants and personnel
Blinding of outcome assessment (detection bias)	Low risk	"Echocardiography was performed before surgery by a single echocardiographer in a blinded fashion."
Incomplete outcome data (attrition bias)	Low risk	All data reported
Selective reporting (reporting bias)	Low risk	All outcomes stated in the methods section were adequately reported or explained in results
Other bias	Unclear risk	Funding for trial: not reported<br>Notable conflicts of interest of authors: not reported

Polymeropoulos 2015

Bias	Authors' judgement	Support for judgement
Random sequence generation (selection bias)	Unclear risk	Insufficient information to form judgement
Allocation concealment (selection bias)	Unclear risk	Insufficient information to form judgement
Blinding of participants and personnel (performance bias)	Unclear risk	Insufficient information to form judgement
Blinding of outcome assessment (detection bias)	Unclear risk	Insufficient information to form judgement
Incomplete outcome data (attrition bias)	Low risk	All data reported
Selective reporting (reporting bias)	Unclear risk	Insufficient information to form judgement
Other bias	Unclear risk	Funding for trial: not reported<br>Notable conflicts of interest of authors: not reported

Sadeghpour 2015

**Bias**	**Authors' judgement**	**Support for judgement**
Random sequence generation (selection bias)	Low risk	"The study population was randomized one day before surgery to two groups (by using www.randomaizer.org). The method of randomization was balanced block with an allocation sequence based on a block size of eight, generated with a computer random number generator."
Allocation concealment (selection bias)	Low risk	"The study population was randomized one day before surgery to two groups (by using www.randomaizer.org). The method of randomization was balanced block with an allocation sequence based on a block size of eight, generated with a computer random number generator."
Blinding of participants and personnel (performance bias)	Low risk	"Both the patients and the hospital staff were blind to the treatment allocation."
Blinding of outcome assessment (detection bias)	Unclear risk	Insufficient information to form judgement
Incomplete outcome data (attrition bias)	Low risk	All data reported
Selective reporting (reporting bias)	Low risk	All outcomes stated in the methods section were adequately reported or explained in results
Other bias	Unclear risk	Funding for trial: not reported<br>Notable conflicts of interest of authors: not reported

Safaei 2017

Bias	Authors' judgement	Support for judgement
Random sequence generation (selection bias)	Low risk	"Patients were randomly assigned to three groups (n = 29 each) using random allocation software."
Allocation concealment (selection bias)	Low risk	"Patients were randomly assigned to three groups (n = 29 each) using random allocation software."
Blinding of participants and personnel (performance bias)	High risk	No blinding of participants and personnel.
Blinding of outcome assessment (detection bias)	Low risk	"All data were collected by an independent research nurse assigned to this research study and were blinded to the groups."<br>"All clinical data were collected by an independent end-point assessor team including a cardiologist and a nurse who were assigned to this clinical trial and were blinded to group assignment."
Incomplete outcome data (attrition bias)	Low risk	Less than 20% lost to follow-up
Selective reporting (reporting bias)	Low risk	All outcomes stated in the methods section were adequately reported or explained in results
Other bias	Unclear risk	Funding for trial: Cardiovascular Research Center, Tabriz University of<br>Medical Sciences, Tabriz, Iran<br>Notable conflicts of interest of authors: all authors report no conflict of interest

Sarzaeem 2014

Bias	Authors' judgement	Support for judgement
Random sequence generation (selection bias)	Unclear risk	Insufficient information to form judgement, study reported in Farsi, translation difficult
Allocation concealment (selection bias)	Unclear risk	Insufficient information to form judgement, study reported in Farsi, translation difficult
Blinding of participants and personnel (performance bias)	Unclear risk	Insufficient information to form judgement, study reported in Farsi, translation difficult
Blinding of outcome assessment (detection bias)	Unclear risk	Insufficient information to form judgement, study reported in Farsi, translation difficult
Incomplete outcome data (attrition bias)	Unclear risk	Insufficient information to form judgement, study reported in Farsi, translation difficult
Selective reporting (reporting bias)	Unclear risk	Insufficient information to form judgement, study reported in Farsi, translation difficult
Other bias	Unclear risk	Insufficient information to form judgement, study reported in Farsi, translation difficult

van Wagoner 2003

Bias	Authors' judgement	Support for judgement
Random sequence generation (selection bias)	Unclear risk	Insufficient information to form judgement
Allocation concealment (selection bias)	Unclear risk	Insufficient information to form judgement
Blinding of participants and personnel (performance bias)	Unclear risk	Insufficient information to form judgement
Blinding of outcome assessment (detection bias)	Unclear risk	Insufficient information to form judgement
Incomplete outcome data (attrition bias)	Unclear risk	Insufficient information to form judgement
Selective reporting (reporting bias)	Unclear risk	Insufficient information to form judgement
Other bias	Unclear risk	Funding for trial: not reported<br>Notable conflicts of interest of authors: all authors report no conflict of interest
